# Clinical, regional, and genetic characteristics of Covid-19 patients from UK Biobank

**DOI:** 10.1371/journal.pone.0241264

**Published:** 2020-11-17

**Authors:** David A. Kolin, Scott Kulm, Paul J. Christos, Olivier Elemento

**Affiliations:** 1 The Meyer Cancer Center, Weill Cornell Medicine, Caryl and Israel Englander Institute for Precision Medicine, New York, NY, United States of America; 2 Department of Physiology and Biophysics, Weill Cornell Medicine, New York, NY, United States of America; 3 Department of Population Health Sciences, Weill Cornell Medicine, New York, NY, United States of America; University of Oxford, UNITED KINGDOM

## Abstract

**Background:**

Coronavirus disease 2019 (Covid-19) has rapidly infected millions of people worldwide. Recent studies suggest that racial minorities and patients with comorbidities are at higher risk of Covid-19. In this study, we analyzed the effects of clinical, regional, and genetic factors on Covid-19 positive status.

**Methods:**

The UK Biobank is a longitudinal cohort study that recruited participants from 2006 to 2010 from throughout the United Kingdom. Covid-19 test results were provided to UK Biobank starting on March 16, 2020. The main outcome measure in this study was Covid-19 positive status, determined by the presence of any positive test for a single individual. Clinical risk factors were derived from UK Biobank at baseline, and regional risk factors were imputed using census features local to each participant’s home zone. We used robust adjusted Poisson regression with clustering by testing laboratory to estimate relative risk. Blood types were derived using genetic variants rs8176719 and rs8176746, and genomewide tests of association were conducted using logistic-Firth hybrid regression.

**Results:**

This prospective cohort study included 397,064 UK Biobank participants, of whom 968 tested positive for Covid-19. The unadjusted relative risk of Covid-19 for Black participants was 3.66 (95% CI 2.83–4.74), compared to White participants. Adjusting for Townsend deprivation index alone reduced the relative risk to 2.44 (95% CI 1.86–3.20). Comorbidities that significantly increased Covid-19 risk included chronic obstructive pulmonary disease (adjusted relative risk [ARR] 1.64, 95% CI 1.18–2.27), ischemic heart disease (ARR 1.48, 95% CI 1.16–1.89), and depression (ARR 1.32, 95% CI 1.03–1.70). There was some evidence that angiotensin converting enzyme inhibitors (ARR 1.48, 95% CI 1.13–1.93) were associated with increased risk of Covid-19. Each standard deviation increase in the number of total individuals living in a participant’s locality was associated with increased risk of Covid-19 (ARR 1.14, 95% CI 1.08–1.20). Analyses of genetically inferred blood types confirmed that participants with type A blood had increased odds of Covid-19 compared to participants with type O blood (odds ratio [OR] 1.16, 95% CI 1.01–1.33). A meta-analysis of genomewide association studies across ancestry groups did not reveal any significant loci. Study limitations include confounding by indication, bias due to limited information on early Covid-19 test results, and inability to accurately gauge disease severity.

**Conclusions:**

When assessing the association of Black race with Covid-19, adjusting for deprivation reduced the relative risk of Covid-19 by 33%. In the context of sociological research, these findings suggest that discrimination in the labor market may play a role in the high relative risk of Covid-19 for Black individuals. In this study, we also confirmed the association of blood type A with Covid-19, among other clinical and regional factors.

## Introduction

The coronavirus disease 2019 (Covid-19) pandemic, caused by severe acute respiratory syndrome 2 (SARS-CoV-2), has affected millions of people worldwide. At the onset of the pandemic, it became apparent that Covid-19 was disproportionately afflicting minority populations, and Black individuals were being particularly affected [1–4]. The risk of death from Covid-19 for Black Americans is 92.3 per 100,000 individuals, while for White Americans, risk of death is 45.2 per 100,000 individuals. The association between Black race and Covid-19 is not unique to the United States. In the United Kingdom, the Office for National Statistics found that Black individuals in England and Wales were over fourfold more likely to die from Covid-19 than White individuals [5]. After adjusting for several confounding socioeconomic factors, Black individuals remained at twofold increased risk in that study.

Current evidence indicates that age, male sex, high body-mass index, and comorbidities–including diabetes and hypertension–are also associated with increased risk of Covid-19 [6, 7] For diabetic patients, the combination of increased angiotensin converting enzyme 2 (ACE2), increased furin, impaired T-cell function, and increased interleukin-6 may increase disease severity [8]. For patients with hypertension, differential expression of ACE2 may modulate disease risk [9]. Because coronaviruses use ACE2 to gain entry into cells, at the beginning of the pandemic, concerns arose regarding medications targeting the angiotensin system. However, some evidence suggests that angiotensin converting enzyme inhibitors and angiotensin II receptor blockers do not increase the risk of Covid-19 [10, 11]. Not only do individual characteristics modulate disease risk, but the local environment also affects the likelihood of Covid-19. Regional factors associated with risk of death from Covid-19 include commuting by public transportation, average winter temperatures, and median household income [12].

Recent evidence also suggests that genetics may influence susceptibility to Covid-19. A twins study estimated that total heritability of Covid-19 risk was 50% (95% CI 29–70%) [13]. Analyses of host genomes indicate that ACE2 and OR4C5 gene variants may impact severity of Covid-19 [14, 15]. OR4C5 is a pseudogene in the olfactory receptor loci region that codes for a G-protein-coupled receptor arising from a single-coding exon gene. The Severe Covid-19 GWAS Group recently found an association between the 3p21.31 gene cluster and Covid-19 with respiratory failure [16]. One gene within the cluster, SLC6A20, may increase Covid-19 susceptibility by encoding sodium-imino acid (proline) transporter 1 (SIT1), a protein that interacts with ACE2.

Despite growing knowledge about both the clinical and genetic factors associated with Covid-19 risk, there is a need for additional studies in order to further elucidate the multitude of factors that influence Covid-19 susceptibility [17, 18]. Our study aims to address gaps in current knowledge by analyzing clinical, regional, and genetic risk factors within the UK Biobank, one of the largest, most comprehensive cohorts currently available. In order to assess the association of risk factors with Covid-19, we created adjusted models and conducted genomewide tests of association. The extensive phenotype data contained in the UK Biobank allows for statistical adjustments that are not possible in other cohorts. Our study confirms some previously-observed findings and uncovers several novel associations.

## Materials and methods

### Study design

The UK Biobank review board approved this study. This is an analysis of a single large prospective cohort study from the United Kingdom. The details of patient recruitment and data collection are publicly available. Data were analyzed anonymously.

This prospective cohort study included a population of 397,064 participants from England between the ages of 40 and 69 recruited from 2006 to 2010. While the UK Biobank does include participants from Scotland and Wales, these participants were excluded because testing laboratories were only located in England. Data on baseline characteristics were obtained during an in-person interview at recruitment, and health outcome data were obtained prospectively from electronic health records.

Recently, Public Health England began providing UK Biobank with information on participants’ Covid-19 test results from March 16, 2020 onwards. Covid-19 data included specimen date, specimen type, processing laboratory, inpatient status, and test results. Most UK testing performed during this period was completed in-hospital. Samples were categorized as inpatient if they were retrieved from an emergency care provider, an inpatient location, or if the sample was related to healthcare associated infection. Samples obtained from patients were kept on a medium salt solution during transfer to a testing facility, where samples were tested for Covid-19 using polymerase chain reaction. Approximately four days elapsed between the time of sample retrieval and the time of data transfer to Public Health England.

Clinical risk factors for each participant were extracted at baseline. Regional risk factors were ascribed to each individual by utilizing each participant’s home location, accurate to one kilometer. Regional information was derived from the national census recorded in 2011, specific to each Lower Layer Super Output Areas for England [19]. Genotypic data was sequenced with the Affymetrix UK Biobank Axiom array, with general imputation completed with IMPUTE2 and HLA-specific imputation completed with HLA*IMP:02 [20, 21]. Quality control of genotypic data excluded individuals who were reported as outliers in heterozygosity, contained sex chromosome aneuploidy, had excess relatives in the cohort, and held a genotyping missing rate above 0.1. The variants analyzed were bi-allelic, with a minor allele frequency above 0.001. Written informed consent was obtained from all study participants.

### Study outcome

The main study outcome was any positive test for a single individual. In order to better understand risk factors for inpatient and outpatient Covid-19, we also conducted a nested case-control study within the population of patients that tested positive for Covid-19 on at least one occasion. Patients were categorized as inpatient cases if they had any Covid-19 testing sample marked as inpatient. Any patient without an inpatient sample was considered an outpatient control.

### Statistical analyses

We then calculated the association of clinical, regional, and genetic factors with Covid-19 positive status. Frequencies and percentages, along with means and standard deviations, were calculated for clinical factors for each of the three following categories: all participants, participants with only negative tests, and participants with a minimum of one positive test. We used Poisson regression, with robust standard errors and clustering by laboratory, to estimate risk ratios for Covid-19 positive status [22]. Uncertainty of each laboratory’s final date of testing prevented the use of hazard ratios. Adjusted estimates accounted for age, sex, body-mass index, systolic blood pressure, Townsend deprivation index, and race. Sensitivity analyses with quasi-Poisson models, correcting for overdispersion, generated nearly identical results. Risk ratios for age, body-mass index, and systolic blood pressure were calculated by determining the change in risk of Covid-19 positive status for each ten unit increase in the risk factor.

Analyses of regional demographics were conducted by similarly adjusted risk models, where the risk ratio indicated the change in risk of positive Covid-19 status for each standard deviation increase in the regional risk factor. In order to avoid over-adjustment due to collinearity, when analyzing regional factors, we did not adjust for Townsend deprivation index, which utilizes local census features.

For analyses of genetic associations, we first analyzed the association of ABO blood types with Covid-19 positive status. We then conducted genomewide tests of association to identify novel genetic variants associated with Covid-19. ABO blood types were inferred from the variants rs8176719 and rs8176746, as previously described [23]. Odds ratios of positive Covid-19 status for the inferred blood types were calculated using logistic regression, adjusted for age, sex, body-mass index, race, and Townsend deprivation index. Genomewide tests of association for positive Covid-19 status were calculated using logistic Firth hybrid regression adjusted for age, sex, and the first ten genetic principal components. These tests were performed on samples of either European, Asian, or African ancestry. These samples were determined by applying k-means clustering to the top 40 genetic principal components, identifying clusters based on overlap with reported race, and removing outliers (distance to cluster center greater than the 99^th^ percentile of all distances). The results of the ancestry-specific genomewide association studies were meta-analyzed through weighted Z-scores, as implemented in the PLINK program [24, 25]. For the genomewide analysis, we used the common P value threshold of 5x10^-8^ to determine statistical significance. Pre-specified analyses of the HLA region were conducted using Bonferroni-corrected P value thresholds based on the number of alleles analyzed. Odds ratios of inpatient status for clinical, regional, and genetic analyses were recalculated for the nested population of Covid-19 positive participants. Ninety-five percent confidence intervals (95% CI) were calculated for all effect parameters of interest to assess the precision of the obtained estimates. All analyses were performed in R Version 4.0 (R Foundation for Statistical Computing, Vienna, Austria).

### Ethical approval

This study was approved by Weill Cornell Medical College and UK Biobank. The UK Biobank is overseen by an independent advisory committee, and the data used within this analysis was accessed through the approved application #47137.

## Results

Among 397,064 participants, there were 4,811 (1.2%) tests for Covid-19 in the most recent UK Biobank Covid-19 release (May 18, 2020), of which 1,868 (38.8%) were inpatient (S1 and S2 Figs in S1 Appendix). A total of 1,623 (33.7%) tests were positive, and one participant was tested 20 times. Although, Public Health England notes that duplicate tests may be present in this study due to the arrival of results via several different routes. The majority of tests were upper respiratory tract swabs (31.1%), nasal swabs (16.1%), or nose and throat swabs (12.3%). Of the patients tested, 968 (31.1%) tested positive at least one time. The mean age (± standard deviation) of Covid-19 positive participants was 57.0 ± 9.1 years, 521 (53.8%) were male, and mean body-mass index was 29.1 ± 5.5 kg/m^2^ (S1-S8 Tables in S1 Appendix).

**Fig 1:**
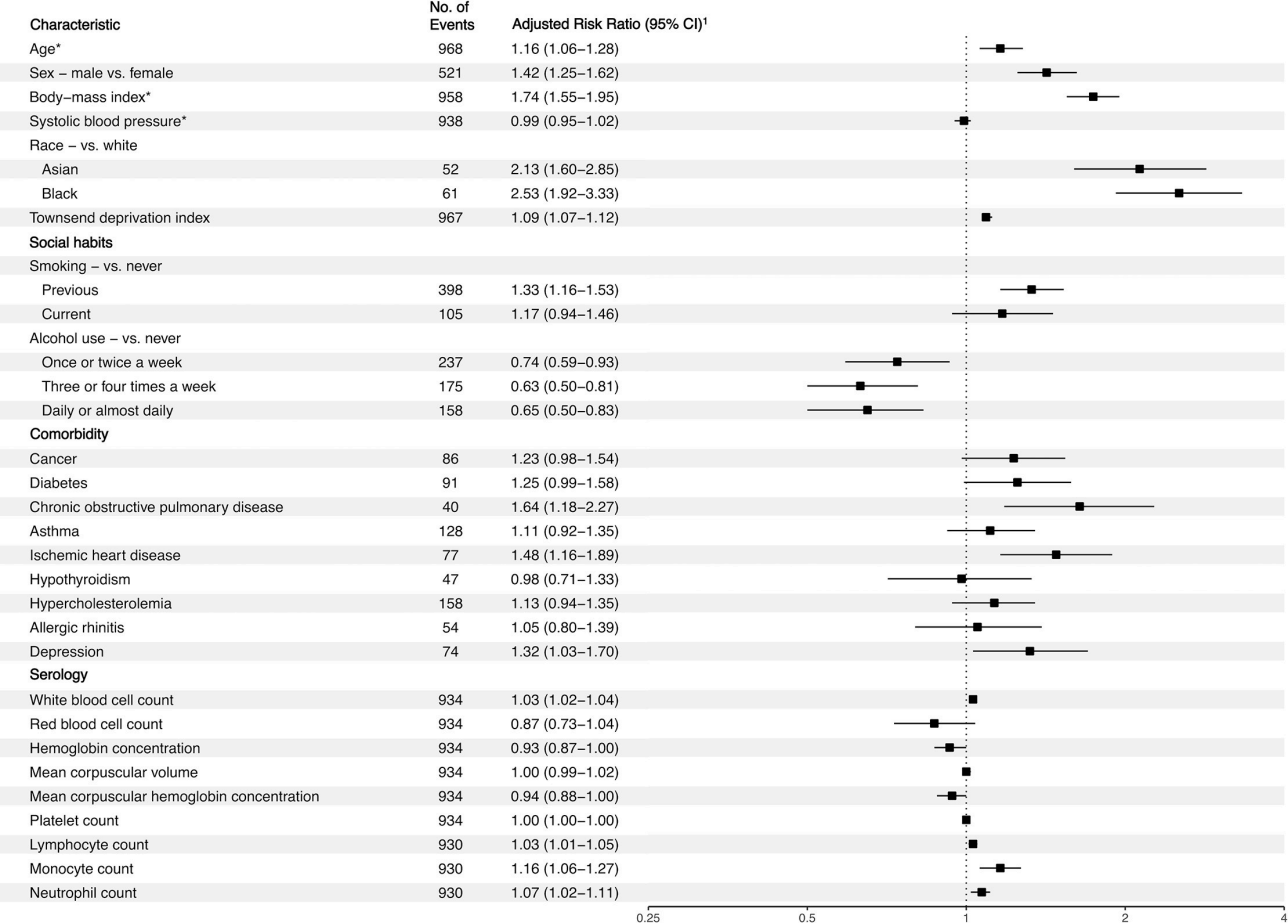
Adjusted risk ratio for Covid-19 positive status with baseline attributes, social habits, comorbidities, and serology. *Adjusted relative risk represents a ten-unit increase in the risk factor. All other risk ratios for continuous variables are for single unit increases in the risk factor. ^1^Risk ratios were adjusted for age, sex, body-mass index, systolic blood pressure, Townsend deprivation index, and race.

Black participants were disproportionately Covid-19 positive. The unadjusted relative risk of Covid-19 for Black participants was 3.66 (95% CI 2.83–4.74) relative to White participants. After only adjusting for deprivation, the relative risk was 2.44 (95% CI 1.86–3.20). After adjusting for age, sex, body-mass index, systolic blood pressure, and Townsend deprivation index, Black participants remained at over two times increased risk of testing positive for Covid-19 compared to White participants (adjusted relative risk [ARR] 2.53, 95% CI 1.92–3.33) (Fig 1). Amongst the 61 Black participants who tested positive for Covid-19, 7 (11.5%) had diabetes and 3 (4.9%) had ischemic heart disease. In exploratory analyses also adjusting for history of diabetes or ischemic heart disease, Black participants remained at increased risk of Covid-19 (ARR 2.54, 95% CI 1.92–3.34) compared to White participants. Asian participants were also at increased risk of testing positive for Covid-19 compared to White participants (ARR 2.13, 95% CI 1.60–2.85). Analyses of serological markers identified that baseline white blood cell count, lymphocyte count, monocyte, and neutrophil count were positively associated with Covid-19.

Among the participants reporting at least one positive Covid-19 test, 91 (9.4%) had a history of diabetes, 40 (4.1%) had a history of chronic obstructive pulmonary disease (COPD), and 77 (8.0%) had a history of ischemic heart disease. After adjusting for confounding, we found that patients were at increased risk of Covid-19 if they had a history of COPD (ARR 1.64, 95% CI 1.18–2.27) or ischemic heart disease (ARR 1.48, 95% CI 1.16–1.89). Participants with depression were at 32% increased risk of Covid-19 (ARR 1.32, 95% CI 1.03–1.70).

**Fig 2:**
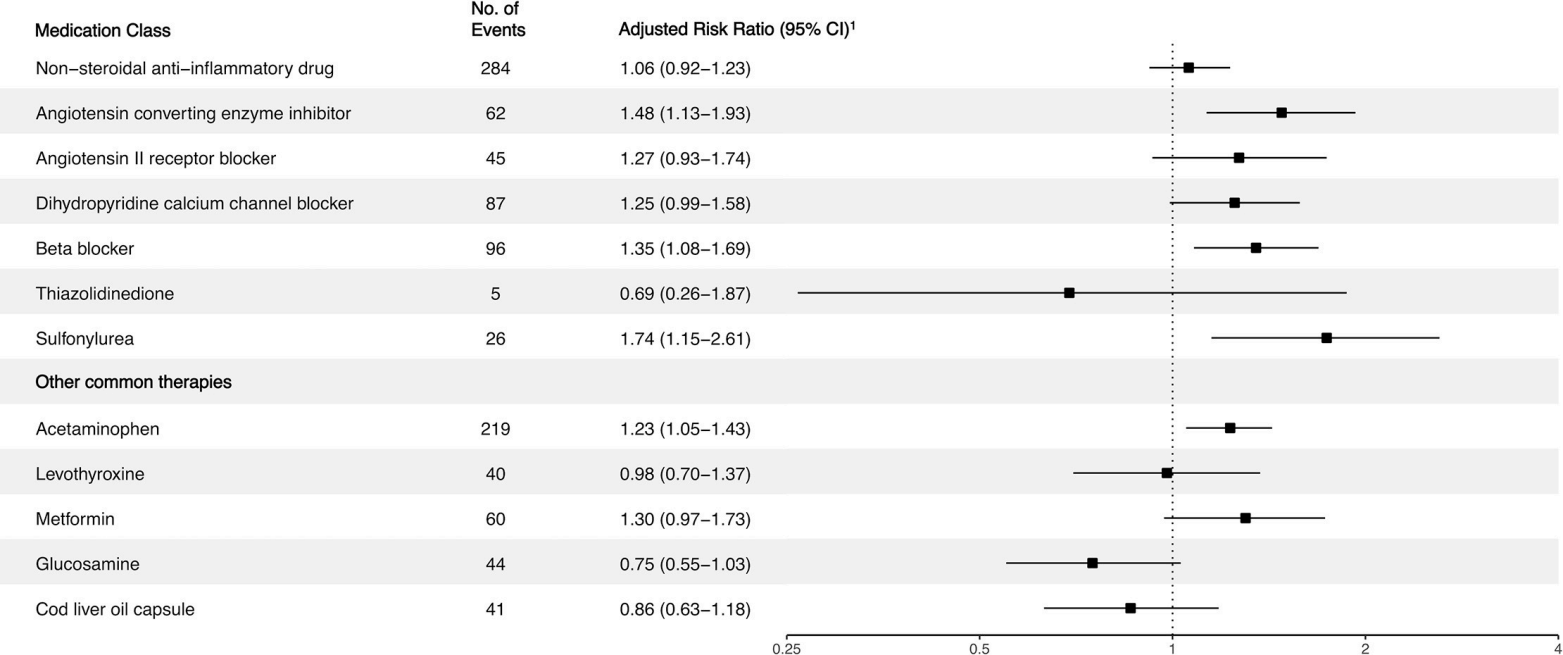
Adjusted risk ratio for Covid-19 positive status with common medications. ^1^Risk ratios were adjusted for age, sex, body-mass index, systolic blood pressure, Townsend deprivation index, and race.

Next, we investigated the association of common classes of medications with Covid-19 (Fig 2). Participants were at increased risk of Covid-19 if they used angiotensin converting enzyme inhibitors (RR 1.48, 95% CI 1.13–1.93) but not angiotensin II receptor blockers (ARR 1.27, 95% CI 0.93–1.74). Of the 61 Black Covid-19 patients, 11 (18.0%) were using either an angiotensin converting enzyme inhibitor or an angiotensin II receptor blocker. Of all Black participants, 494 (6.6%) were using either of the two medications. Adjusted risk ratios for use of non-steroidal anti-inflammatory drugs and acetaminophen were 1.06 (95% CI, 0.92–1.23) and 1.23 (95% CI, 1.05–1.43), respectively. Analyses of Covid-19 positive inpatients and outpatients in the nested case-control study found that the groups were similar across clinical factors.

We then analyzed regional factors, using counts of individuals per census zone, to further understand the effects of the local environment on Covid-19 (S9 and S10 Tables in S1 Appendix). After adjusting for covariates, each standard deviation increase in the number of unemployed individuals was associated with a 19% increased risk of Covid-19, and households with a lone parent in the participant’s home zone was associated with an 18% increased risk. Other regional factors associated with increased Covid-19 risk included the total number of individuals per hectare (ARR 1.14, 95% CI 1.08–1.20), the number of individuals with no academic qualifications (ARR 1.14, 95% CI 1.07–1.21), and the number of individuals who rent their residence (ARR 1.18, 95% CI 1.12–1.24).

**Fig 3:**
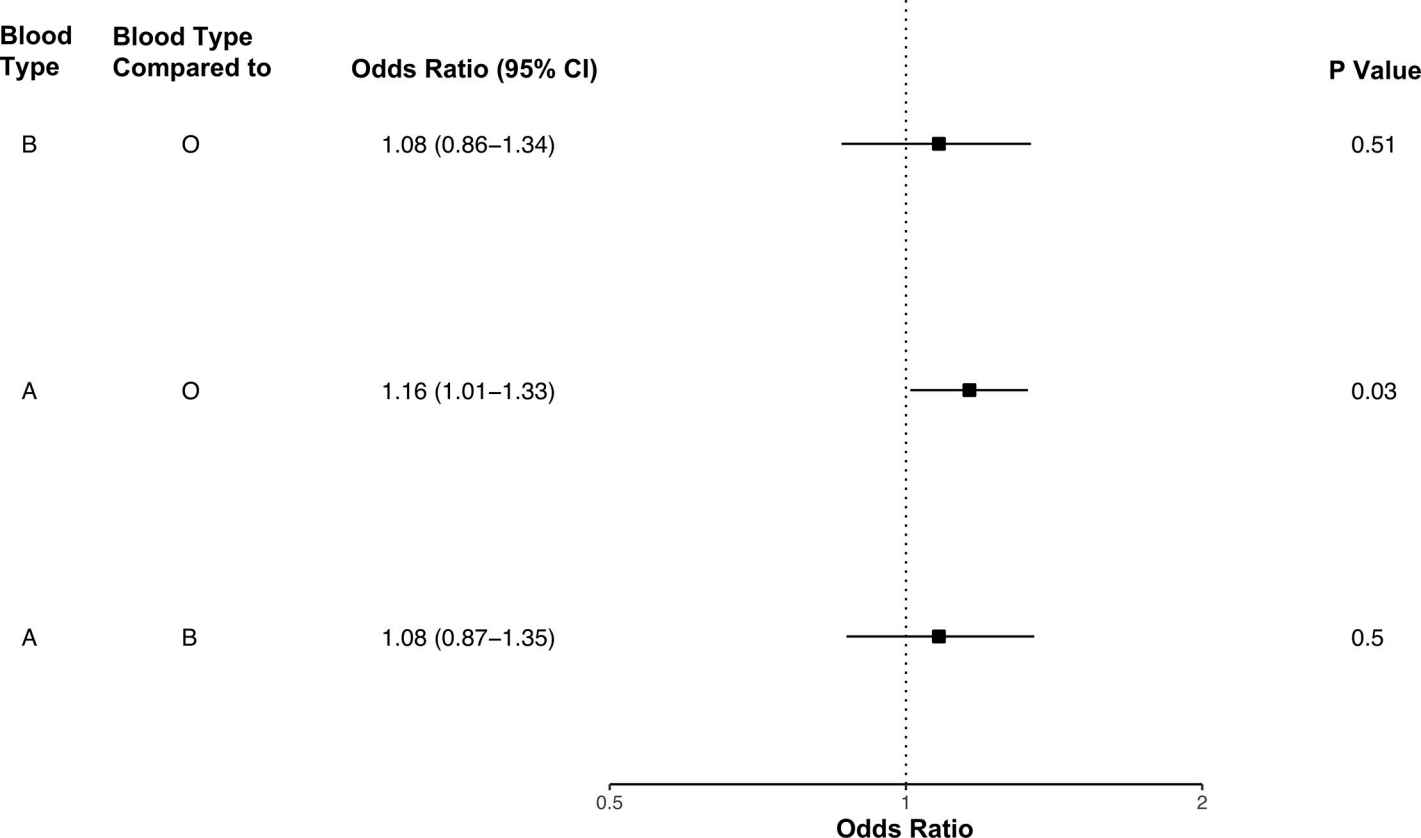
Odds of Covid-19 positive status by blood type.

**Fig 4:**
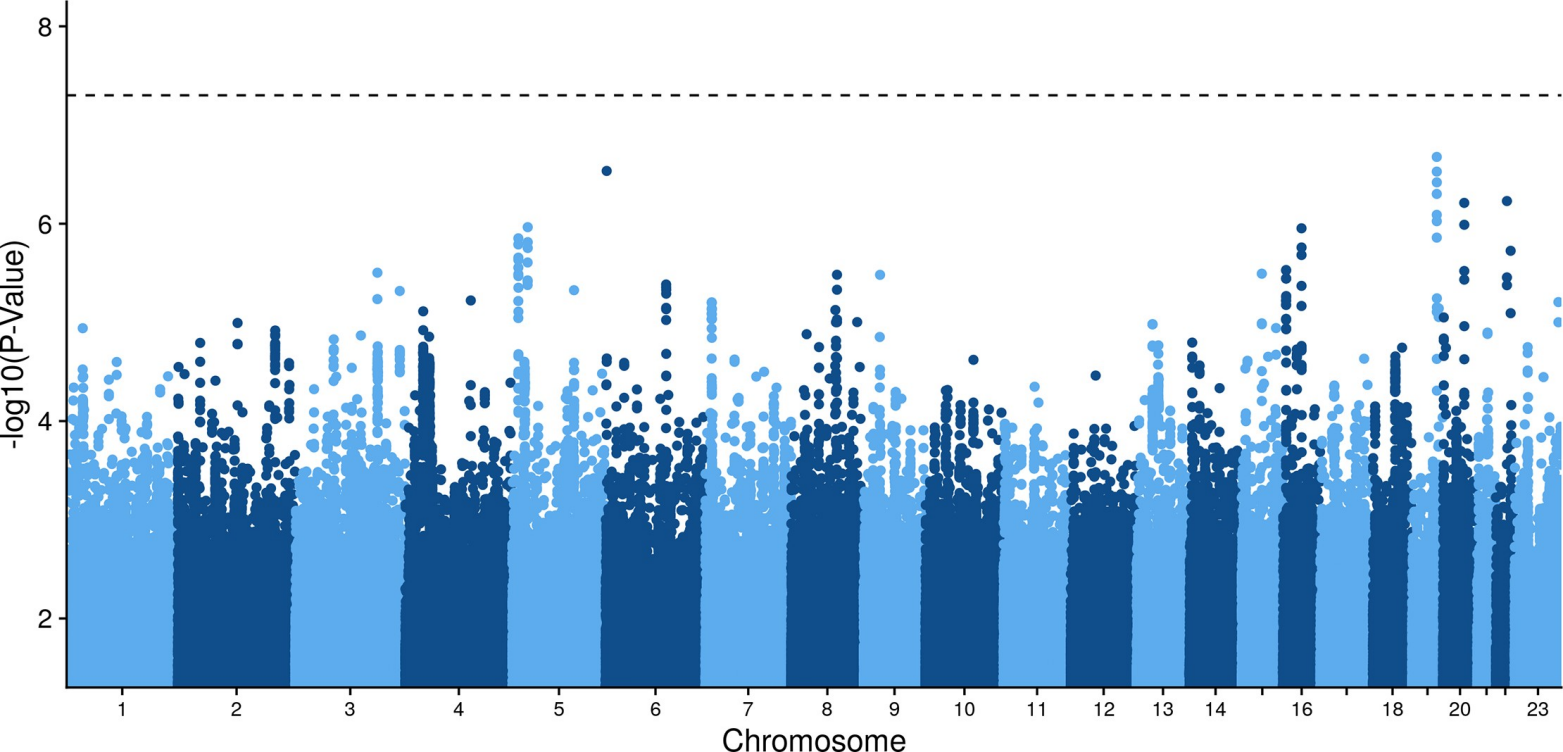
Cross-ancestry genomewide association analysis of Covid-19 positive status.

Lastly, we investigated the genetics of participants with any positive Covid-19 test. ABO blood types of participants were inferred through genetic profiles [23]. Participants with blood type A had increased odds of at least one positive Covid-19 test relative to blood type O participants (odds ratio [OR] 1.16, 95% CI 1.01–1.33) (Fig 3 and S11 Table in S1 Appendix) [26]. Genomewide association analysis of any positive Covid-19 test in the meta-analysis and ancestry specific analyses did not uncover any significant loci (Fig 4). The HLA regional analysis also did not reveal any significant alleles, and analyses of Covid-19 positive inpatients and outpatients identified no significant variants (S3-S9 Figs in S1 Appendix).

## Discussion

In this cohort study of participants from the United Kingdom, we found that the unadjusted risk of Covid-19 was over threefold higher for Black participants than for White participants. Adjusting for deprivation alone reduced the relative risk by 33%, and adjusted effect estimates–accounting for a range of comorbidities–did not alter the relative risk of Covid-19 for Black participants considerably. These novel analyses suggest that differences in deprivation amongst racial groups is one of the greatest drivers of Covid-19 for Black individuals. Our analyses also identified that patients with COPD, ischemic heart disease, and depression were more likely to test positive for Covid-19. Participants in impoverished, populous regions, with high unemployment and low home ownership, were at higher risk of Covid-19. Finally, blood type A was associated with Covid-19 positive status.

Overall, data from recent studies suggest that comorbidities play a significant role in Covid-19 disease severity. A study from two New York City hospitals found that about one quarter of all Covid-19 patients were diabetic, and half of all patients had hypertension [27]. A separate study of 5,700 patients found that the most common comorbidities were hypertension (56.6%), obesity (41.7%), and diabetes (33.8%) [28]. The OpenSAFELY study, which analyzed data from over 17,000,000 patients, found significant associations between Covid-19-related-death and comorbidities, including hypertension, obesity, and diabetes [29]. Additionally, the study found that reduced kidney function (eGFR <30), hematological malignancy, organ transplant, and other neurological disease were associated with over threefold increased risk of Covid-19-related-death. To our knowledge, our study is the first study to suggest that depression is a risk factor for Covid-19. It is well-established that the common comorbidities identified in Covid-19 patients are associated with inflammation [30–32]. In our study, novel serological analyses within the UK Biobank cohort further emphasized the importance of inflammation; increased white blood cell, lymphocyte, monocyte, and neutrophil counts were all associated with increased risk of Covid-19. Higher baseline inflammation may result in more severe Covid-19 and subsequent testing.

This study lends some support to the reported hypothesis that upregulation of ACE2 is a risk factor for Covid-19. After adjustment for several confounding factors, there was an association between medications targeting the angiotensin system and Covid-19 [33, 34]. When considering the totality of current evidence, it is unlikely that medications targeting the angiotensin system increase the likelihood of Covid-19. Rather, residual confounding in this study–due to confounding by indication–may have led to the observed association. Residual confounding likely remained, despite our efforts to control for six confounders at baseline.

The regional factors in this study suggest that impoverished neighborhoods have a greater burden of Covid-19. Participants in this cohort were more likely to test positive for Covid-19 if there were more unemployed individuals, more renters, and more individuals without advanced degrees in their locality. One potential explanation for this finding is that less affluent individuals may not be able to quarantine effectively during the pandemic due to financial limitations and the need to ensure job security. According to analyses of data provided by *Cuebiq*, a company that tracks approximately 15 million cellphone users daily, wealthier individuals began staying home earlier and are moving less than poorer individuals [35]. In light of these findings, additional efforts should be made to safeguard impoverished individuals from infection. In order to reduce viral transmission, employers should provide masks to employees and grant sick leave to employees who are ill (without repercussions) [36].

Recently, a significant association between blood type A and Covid-19 has been identified in multiple different populations, including our own [16, 37]. The GATC haplotype–prevalent in non-O blood type patients–is associated with ACE activity, potentially facilitating SARS-CoV-2 entry into host cells [23]. Although we did not discover any genomewide significant loci in this analysis of the UK Biobank, Ellinghaus et al. reported a single significant locus on 3p21.31, which includes a gene associated with the renin-angiotensin-aldosterone-system (RAAS) pathway. While the association of a RAAS gene with Covid-19 increases causal likelihood, our inability to confirm its significance may underscore its weak effect on Covid-19 susceptibility [38, 39].

Multiple reports both in the United States and the United Kingdom have shown that Covid-19 is disproportionately affecting Black populations [18, 40]. A previous study of the UK Biobank found that participants in the lowest socioeconomic disadvantage quartile were over twice as likely to have a confirmed infection, than those in the highest quartile [41]. In order to understand these effects, we further investigated the effects of adjusting for the Townsend deprivation index alone. When considered as a whole, the findings from this study suggest that racial discrimination may be contributing to the higher likelihood that Black individuals test positive for Covid-19 relative to White individuals [4]. Adjustment only for the Townsend deprivation index–a score that combines household overcrowding, non-home ownership, non-car ownership, and unemployment–led to a relative risk reduction of 33%. Census analyses also found that that unemployment is strongly associated with increased Covid-19 risk.

Numerous randomized sociological studies have confirmed strong associations between race and diminished employment prospects. In the United States, a randomized trial of equivalent job applications with White and Black sounding names led to 50% more callbacks for applicants with White sounding names [42]. Studies in the United Kingdom have led to similar findings [43]. Recently, a randomized controlled trial of nearly 3,200 identical job applications found that, relative to the White British majority, Black British individuals–from Nigeria, Ethiopia, Somalia, and Uganda–had to send twice as many job applications to receive a callback [44]. Differences in response to identical job applications may partially explain why Black participants in this study had greater deprivation, a measure of unemployment, than White participants. Given the strong associations between deprivation, race, and Covid-19 in this study, our findings may be due, in part, to discriminatory hiring practices within the labor market. Finally, any genetic differences amongst races are highly unlikely to explain our results, as multiple studies have shown that genetic variation within racial groups is greater than between racial groups [45–47].

### Limitations

This study has several limitations. We primarily used log-link robust Poisson regression to estimate the relative risk of Covid-19 positive status in this longitudinal cohort. While the effect estimates produced by hazard ratios likely would have been superior to those generated by robust Poisson regression, the calculation of hazard ratios in this study was not possible. While testing center sample reporting to UK Biobank began on March 16, 2020, the final date of testing for each individual center could not be reliably established, as data release to UK Biobank is ongoing. Because inclusion of testing results into UK Biobank did not occur until March 16, 2020, participants may have been included as at-risk, despite having had a positive Covid-19 test result that was not included in the dataset. However, the bias caused by inclusion of early Covid-19 positive patients was likely small because, on March 16, there were only 1,391 confirmed Covid-19 cases in the United Kingdom [48]. Additionally, limitations in the availability of testing likely prevented some participants with severe disease, and many participants with mild disease, from testing positive for Covid-19. It is important to note that the primary outcome in this study was Covid-19 positive status, verified by testing. Many individuals in the cohort likely were positive for Covid-19, without ever having a positive test. While inpatient status was used as a proxy for severe disease, other markers of disease severity–such as degree of hypoxemia and length of intensive care unit stay–were not recorded.

## Conclusions

In this study, we analyzed a cohort of nearly 400,000 participants, 968 of whom tested positive for Covid-19. We found that participants with comorbidities, including COPD, ischemic heart disease, and depression, were at increased risk of Covid-19. We also confirmed the association of Covid-19 with blood type A. In this study, the relative risk of Covid-19 for Black participants, relative to White participants, was reduced by 33% with adjustment for deprivation alone. When assessing differences in risk of Covid-19 between White and Black participants, adjustment for deprivation had a greater effect on the relative risk than any other risk factor analyzed. The increased risk of Covid-19 for Black participants may be linked to discrimination against Black individuals in the labor market, among other factors.

## Supporting information

S1 Appendix (DOCX)

## References

[1] COVID-19 in Racial and Ethnic Minority Groups. Centers for Disease Control and Prevention. 2020.

[2] Webb Hooper M, Nápoles AM, Pérez-Stable EJ. COVID-19 and Racial/Ethnic Disparities. JAMA. 2020 [cited 11 Jun 2020]. 10.1001/jama.2020.8598 32391864 PMC9310097

[3] Price-Haywood EG, Burton J, Fort D, Seoane L. Hospitalization and Mortality among Black Patients and White Patients with Covid-19. New England Journal of Medicine. 2020 [cited 11 Jun 2020]. 10.1056/NEJMsa2011686 32459916 PMC7269015

[4] Laurencin CT, McClinton A. The COVID-19 Pandemic: a Call to Action to Identify and Address Racial and Ethnic Disparities. Journal of Racial and Ethnic Health Disparities. 2020;7: 398–402. 10.1007/s40615-020-00756-0 32306369 PMC7166096

[5] Coronavirus (COVID-19) related deaths by ethnic group, England and Wales: 2 March 2020 to 10 April 2020. Office for National Statistics. 2020. Available: https://www.ons.gov.uk/peoplepopulationandcommunity/birthsdeathsandmarriages/deaths/articles/coronavirusrelateddeathsbyethnicgroupenglandandwales/2march2020to10april2020

[6] Wang B, Li R, Lu Z, Huang Y. Does comorbidity increase the risk of patients with COVID-19: evidence from meta-analysis. Aging. 2020 [cited 11 Jun 2020]. 10.18632/aging.103000 32267833 PMC7185114

[7] Chung RY-N, Dong D, Li MM. Socioeconomic gradient in health and the covid-19 outbreak. BMJ. 2020; m1329 10.1136/bmj.m1329 32238351

[8] Singh AK, Gupta R, Ghosh A, Misra A. Diabetes in COVID-19: Prevalence, pathophysiology, prognosis and practical considerations. Diabetes & Metabolic Syndrome: Clinical Research & Reviews. 2020;14: 303–310. 10.1016/j.dsx.2020.04.004 32298981 PMC7195120

[9] Little P. Non-steroidal anti-inflammatory drugs and covid-19. BMJ. 2020; m1185 10.1136/bmj.m1185 32220865

[10] Mehta N, Kalra A, Nowacki AS, Anjewierden S, Han Z, Bhat P, et al Association of Use of Angiotensin-Converting Enzyme Inhibitors and Angiotensin II Receptor Blockers With Testing Positive for Coronavirus Disease 2019 (COVID-19). JAMA Cardiology. 2020 [cited 20 Jun 2020]. 10.1001/jamacardio.2020.1855 32936273 PMC7201375

[11] Fosbøl EL, Butt JH, Østergaard L, Andersson C, Selmer C, Kragholm K, et al Association of Angiotensin-Converting Enzyme Inhibitor or Angiotensin Receptor Blocker Use With COVID-19 Diagnosis and Mortality. JAMA. 2020 [cited 20 Jun 2020]. 10.1001/jama.2020.11301 32558877 PMC7305566

[12] Knittel C, Ozaltun B. What Does and Does Not Correlate With COVID-19 Death Rates? MIT Center for Energy and Environmental Policy Research. 2020;Working Paper Series.

[13] Williams FM, Freydin M, Mangino M, Couvreur S, Visconti A, Bowyer RC, et al Self-reported symptoms of covid-19 including symptoms most predictive of SARS-CoV-2 infection, are heritable. Genetic and Genomic Medicine; 2020 Apr. 10.1101/2020.04.22.20072124 33558003

[14] Benetti E, Giliberti A, Emiliozzi A, Velentino F, Bergantini L, Fallerini C, et al Clinical and molecular characterization of COVID-19 hospitalized patients. Genetic and Genomic Medicine; 2020 May. 10.1101/2020.05.22.20108845 PMC7673557 33206719

[15] Khayat AS, Assumpcao PP de, Khayat BCM, Araujo TMT, Batista-Gomes JA, Imbiriba LC, et al ACE2 polymorphisms as potential players in COVID-19 outcome. Genetic and Genomic Medicine; 2020 May. 10.1101/2020.05.27.20114843 PMC7769452 33370311

[16] Ellinghaus D, Degenhardt F, Bujanda L, Buti M, Albillos A, Invernizzi P, et al Genomewide Association Study of Severe Covid-19 with Respiratory Failure. New England Journal of Medicine. 2020 [cited 20 Jun 2020]. 10.1056/NEJMoa2020283 32558485 PMC7315890

[17] Khalatbari-Soltani S, Cumming RG, Delpierre C, Kelly-Irving M. Importance of collecting data on socioeconomic determinants from the early stage of the COVID-19 outbreak onwards. Journal of Epidemiology and Community Health. 2020; jech-2020-214297. 10.1136/jech-2020-214297 32385126 PMC7298202

[18] Yancy CW. COVID-19 and African Americans. JAMA. 2020 [cited 18 Apr 2020]. 10.1001/jama.2020.6548 32293639

[19] Nomis Official Labor Market Statistics. Office for National Statistics. 2011. Available: https://www.nomisweb.co.uk/census/2011/key_statistics_uk

[20] Dilthey A, Leslie S, Moutsianas L, Shen J, Cox C, Nelson MR, et al Multi-Population Classical HLA Type Imputation. Browning S, editor. PLoS Computational Biology. 2013;9: e1002877 10.1371/journal.pcbi.1002877 23459081 PMC3572961

[21] Howie BN, Donnelly P, Marchini J. A Flexible and Accurate Genotype Imputation Method for the Next Generation of Genome-Wide Association Studies. Schork NJ, editor. PLoS Genetics. 2009;5: e1000529 10.1371/journal.pgen.1000529 19543373 PMC2689936

[22] McNutt L-A. Estimating the Relative Risk in Cohort Studies and Clinical Trials of Common Outcomes. American Journal of Epidemiology. 2003;157: 940–943. 10.1093/aje/kwg074 12746247

[23] Groot HE, Villegas Sierra LE, Said MA, Lipsic E, Karper JC, van der Harst P. Genetically Determined ABO Blood Group and its Associations With Health and Disease. Arteriosclerosis, Thrombosis, and Vascular Biology. 2020;40: 830–838. 10.1161/ATVBAHA.119.313658 31969017

[24] Chang CC, Chow CC, Tellier LC, Vattikuti S, Purcell SM, Lee JJ. Second-generation PLINK: rising to the challenge of larger and richer datasets. GigaScience. 2015;4 10.1186/s13742-015-0047-8 25722852 PMC4342193

[25] Willer CJ, Li Y, Abecasis GR. METAL: fast and efficient meta-analysis of genomewide association scans. Bioinformatics. 2010;26: 2190–2191. 10.1093/bioinformatics/btq340 20616382 PMC2922887

[26] Zhao J, Yang Y, Huang H, Li D, Gu D, Lu X, et al Relationship between the ABO Blood Group and the COVID-19 Susceptibility. Epidemiology; 2020 Mar. 10.1101/2020.03.11.20031096

[27] Goyal P, Choi JJ, Pinheiro LC, Schenck EJ, Chen R, Jabri A, et al Clinical Characteristics of Covid-19 in New York City. New England Journal of Medicine. 2020 [cited 16 May 2020]. 10.1056/NEJMc2010419 32302078 PMC7182018

[28] Richardson S, Hirsch JS, Narasimhan M, Crawford JM, McGinn T, Davidson KW, et al Presenting Characteristics, Comorbidities, and Outcomes Among 5700 Patients Hospitalized With COVID-19 in the New York City Area. JAMA. 2020 [cited 16 May 2020]. 10.1001/jama.2020.6775 32320003 PMC7177629

[29] Williamson EJ, Walker AJ, Bhaskaran K, Bacon S, Bates C, Morton CE, et al Factors associated with COVID-19-related death using OpenSAFELY. Nature. 2020;584: 430–436. 10.1038/s41586-020-2521-4 32640463 PMC7611074

[30] Tsalamandris S, First Cardiology Clinic, Hippokration General Hospital, National and Kapodistrian University of Athens, School of Medicine, Athens, Greece, Antonopoulos AS, First Cardiology Clinic, Hippokration General Hospital, National and Kapodistrian University of Athens, School of Medicine, Athens, Greece, Oikonomou E, First Cardiology Clinic, Hippokration General Hospital, National and Kapodistrian University of Athens, School of Medicine, Athens, Greece, et al The Role of Inflammation in Diabetes: Current Concepts and Future Perspectives. European Cardiology Review. 2019;14: 50 10.15420/ecr.2018.33.1 31131037 PMC6523054

[31] Tomiyama H, Shiina K, Matsumoto‐Nakano C, Ninomiya T, Komatsu S, Kimura K, et al The Contribution of Inflammation to the Development of Hypertension Mediated by Increased Arterial Stiffness. Journal of the American Heart Association. 2017;6 10.1161/JAHA.117.005729 28666991 PMC5586296

[32] Ellulu MS, Patimah I, Khaza’ai H, Rahmat A, Abed Y. Obesity and inflammation: the linking mechanism and the complications. Archives of Medical Science. 2017;4: 851–863. 10.5114/aoms.2016.58928 28721154 PMC5507106

[33] Fang L, Karakiulakis G, Roth M. Are patients with hypertension and diabetes mellitus at increased risk for COVID-19 infection? The Lancet Respiratory Medicine. 2020;8: e21 10.1016/S2213-2600(20)30116-8 32171062 PMC7118626

[34] Tignanelli CJ, Ingraham NE, Sparks MA, Reilkoff R, Bezdicek T, Benson B, et al Antihypertensive drugs and risk of COVID-19? The Lancet Respiratory Medicine. 2020 [cited 18 Apr 2020]. 10.1016/S2213-2600(20)30153-3 32222166 PMC7194709

[35] Valentino-Devries J, Lu D, Dance G. Location Data Says it All: Staying at Home During Coronavirus Is a Luxury. The New York Times. 2020 Available: https://www.nytimes.com/interactive/2020/04/03/us/coronavirus-stay-home-rich-poor.html

[36] Dyal JW, Grant MP, Broadwater K, Bjork A, Waltenburg MA, Gibbins JD, et al COVID-19 Among Workers in Meat and Poultry Processing Facilities ― 19 States, April 2020. MMWR Morbidity and Mortality Weekly Report. 2020; doi: 10.15585/mmwr.mm6918e3 32379731 10.15585/mmwr.mm6918e3

[37] Zietz M, Tatonetti NP. Testing the association between blood type and COVID-19 infection, intubation, and death. medRxiv. 2020 10.1101/2020.04.08.20058073 33188185 PMC7666188

[38] Khadjeh S, Hindmarsh V, Weber F, Cyganek L, Vidal RO, Torkieh S, et al CRISPLD1: a novel conserved target in the transition to human heart failure. Basic Research in Cardiology. 2020;115 10.1007/s00395-020-0784-4 32146539 PMC7060963

[39] Wang J-Y, Zhang Y-J, Li H, Hu X-L, Li M-P, Song P-Y, et al CRISPLD1 rs12115090 polymorphisms alters antiplatelet potency of clopidogrel in coronary artery disease patients in Chinese Han. Gene. 2018;678: 226–232. 10.1016/j.gene.2018.08.027 30096456

[40] Khunti K. Is ethnicity linked to incidence or outcomes of covid-19? BMJ. 2020. 10.1136/bmj.m1548 32312785

[41] Niedzwiedz CL, O’Donnell CA, Jani BD, Demou E, Ho FK, Celis-Morales C, et al Ethnic and socioeconomic differences in SARS-CoV-2 infection: prospective cohort study using UK Biobank. BMC Medicine. 2020;18 10.1186/s12916-020-01640-8 32466757 PMC7255908

[42] Bertrand M, Mullainathan S. Are Emily and Greg More Employable Than Lakisha and Jamal? A Field Experiment on Labor Market Discrimination. American Economic Review. 2004;94: 991–1013. 10.1257/0002828042002561

[43] Heath AF, Di Stasio V. Racial discrimination in Britain, 1969–2017: a meta‐analysis of field experiments on racial discrimination in the British labour market. The British Journal of Sociology. 2019;70: 1774–1798. 10.1111/1468-4446.12676 31168788

[44] Di Stasio V, Heath A. Are employers in Britain discriminating against ethnic minorities? Centre for Social Investigation. 2019; 1–10.

[45] Vyas DA, Eisenstein LG, Jones DS. Hidden in Plain Sight—Reconsidering the Use of Race Correction in Clinical Algorithms. Malina D, editor. New England Journal of Medicine. 2020 [cited 24 Jun 2020]. 10.1056/NEJMms2004740 32853499

[46] Maglo KN, Mersha TB, Martin LJ. Population Genomics and the Statistical Values of Race: An Interdisciplinary Perspective on the Biological Classification of Human Populations and Implications for Clinical Genetic Epidemiological Research. Frontiers in Genetics. 2016;7 10.3389/fgene.2016.00007 26925096 PMC4756148

[47] Fuentes A, Ackermann RR, Athreya S, Bolnick D, Lasisi T, Lee S, et al AAPA Statement on Race and Racism. American Journal of Physical Anthropology. 2019;169: 400–402. 10.1002/ajpa.23882 31199004

[48] Rosner M, Ritchie H, Ortiz-Ospina E, Hasell J. Statistics and Research: Coronavirus (COVID-19) Cases. Our World in Data. 2020 Available: https://ourworldindata.org/covid-cases?country=~GBR#acknowledgments

