# Supplementary material for: Clinical, regional, and genetic characteristics of Covid-19 patients from UK Biobank
Source: PLoS One. 2020 Nov 17;15(11):e0241264. doi: 10.1371/journal.pone.0241264 (PMC7671499; doi:10.1371/journal.pone.0241264)
Supplement: S1 Appendix — (DOCX) [file pone.0241264.s001.docx]

**S1 Appendix**

CONTENTS

| Supplementary Table 1 | Page 2 |
| --- | --- |
| Supplementary Table 2 | Page 4 |
| Supplementary Table 3 | Page 5 |
| Supplementary Table 4 | Page 7 |
| Supplementary Table 5 | Page 8 |
| Supplementary Table 6 | Page 10 |
| Supplementary Table 7 | Page 11 |
| Supplementary Table 8 | Page 12 |
| Supplementary Table 9 | Page 13 |
| Supplementary Table 10 | Page 15 |
| Supplementary Table 11 | Page 17 |
| Supplementary Figure 1 | Page 18 |
| Supplementary Figure 2 | Page 19 |
| Supplementary Figure 3 | Page 20 |
| Supplementary Figure 4 | Page 21 |
| Supplementary Figure 5  Supplementary Figure 6  Supplementary Figure 7  Supplementary Figure 8  Supplementary Figure 9 | Page 22  Page 23  Page 24  Page 25  Page 26 |
|  |  |

**S1 Table.** Baseline Characteristics of UK Biobank Participants

| **Characteristic** | **All Participants**  **(N = 397064)** | **All Tests Negative**  **(N = 1734)** | **At Least One Positive Test**  **(N = 968)** |
| --- | --- | --- | --- |
| **Baseline and demographic** |  |  |  |
| Age, mean (SD), y | 56.4 (8.1) | 57.1 (8.9) | 57.0 (9.1) |
| Male, No. (%) | 179353 (45.2) | 799 (46.1) | 521 (53.8) |
| Body-mass index, mean (SD)* | 27.4 (4.7) | 28.2 (5.5) | 29.1 (5.5) |
| Systolic blood pressure, mean (SD), mmHg | 139.6 (19.6) | 139.4 (20.2) | 140.9 (20.7) |
| Race, No. (%) |  |  |  |
| White | 374090 (94.2) | 1608 (92.7) | 839 (86.7) |
| Asian | 10181 (2.6) | 42 (2.4) | 52 (5.4) |
| Black | 7431 (1.9) | 53 (3.1) | 61 (6.3) |
| Townsend deprivation index, mean (SD) | -1.4 (3.0) | -0.6 (3.3) | -0.2 (3.5) |
| **Social habit** |  |  |  |
| Smoking, No. (%) |  |  |  |
| Never | 219005 (55.2) | 833 (48.0) | 455 (47.0) |
| Previous | 137024 (34.5) | 654 (37.7) | 398 (41.1) |
| Current | 39023 (9.8) | 238 (13.7) | 105 (10.8) |
| Alcohol use, No. (%) |  |  |  |
| Never | 30580 (7.7) | 173 (10.0) | 126 (13.0) |
| Once or twice a week | 102010 (25.7) | 408 (23.5) | 237 (24.5) |
| Three or four times a week | 92661 (23.3) | 342 (19.7) | 175 (18.1) |
| Daily or almost daily | 81720 (20.6) | 361 (20.8) | 158 (16.3) |
| **Comorbidity** |  |  |  |
| Cancer, No. (%) | 30975 (7.8) | 183 (10.6) | 86 (8.9) |
| Diabetes, No. (%) | 19494 (4.9) | 156 (9.0) | 91 (9.4) |
| Chronic obstructive pulmonary disease, No. (%)^1^ | 8557 (2.2) | 73 (4.2) | 40 (4.1) |
| Asthma, No. (%) | 45968 (11.6) | 245 (14.1) | 128 (13.2) |
| Ischemic heart disease, No. (%)^2^ | 16317 (4.1) | 131 (7.6) | 77 (8.0) |
| Hypothyroidism, No. (%) | 19356 (4.9) | 77 (4.4) | 47 (4.9) |
| Hypercholesterolemia, No. (%) | 48380 (12.2) | 284 (16.4) | 158 (16.3) |
| Allergic rhinitis, No. (%) | 23644 (6.0) | 96 (5.5) | 54 (5.6) |
| Depression, No. (%) | 22319 (5.6) | 151 (8.7) | 74 (7.6) |
| **Serology** |  |  |  |
| White blood cell count, mean (SD) | 6.9 (2.0) | 7.1 (2.0) | 7.3 (4.4) |
| Red blood cell count, mean (SD) | 4.5 (0.4) | 4.5 (0.4) | 4.6 (0.4) |
| Hemoglobin concentration, mean (SD) | 14.2 (1.2) | 14.1 (1.3) | 14.2 (1.4) |
| Mean corpuscular volume, mean (SD) | 91.0 (4.5) | 91.2 (5.0) | 90.7 (5.3) |
| Mean corpuscular hemoglobin concentration, mean (SD) | 34.5 (1.1) | 34.4 (1.0) | 34.4 (1.0) |
| Platelet count, mean (SD) | 252.3 (59.2) | 251.6 (61.1) | 250.1 (63.1) |
| Lymphocyte count, mean (SD) | 2.0 (1.1) | 2.0 (0.9) | 2.1 (4.0) |
| Monocyte count, mean (SD) | 0.5 (0.2) | 0.5 (0.2) | 0.5 (0.3) |
| Neutrophil count, mean (SD) | 4.2 (1.4) | 4.4 (1.5) | 4.4 (1.5) |

*Body-mass index is the weight in kilograms divided by the square of the height in meters.

^1^Chronic obstructive pulmonary disease was defined as a diagnosis of emphysema and/or bronchitis.

^2^Ischemic heart disease was categorized as history of myocardial infarction or angina.

**S2 Table.** Medication Use of UK Biobank Participants and Covid-19 Status

| **Medication Class** | **All Participants**  **(N = 397064)** | **All Tests Negative**  **(N = 1734)** | **At Least One Positive Test**  **(N = 968)** |
| --- | --- | --- | --- |
| Non-steroidal anti-inflammatory drug, No. (%)^1^ | 102748 (25.9) | 546 (31.5) | 284 (29.3) |
| Angiotensin converting enzyme inhibitor, No. (%)^2^ | 13677 (3.4) | 83 (4.8) | 62 (6.4) |
| Angiotensin II receptor blocker, No. (%)^3^ | 11138 (2.8) | 81 (4.7) | 45 (4.6) |
| Dihydropyridine calcium channel blocker, No. (%)^4^ | 20886 (5.3) | 148 (8.5) | 87 (9.0) |
| Beta blocker, No. (%)^5^ | 23670 (6.0) | 140 (8.1) | 96 (9.9) |
| Thiazolidinedione, No. (%)^6^ | 1367 (0.3) | 13 (0.7) | 5 (0.5) |
| Sulfonylurea, No. (%)^7^ | 3625 (0.9) | 46 (2.7) | 26 (2.7) |
| **Other Common Therapies** |  |  |  |
| Acetaminophen, No. (%) | 72593 (18.3) | 389 (22.4) | 219 (22.6) |
| Levothyroxine, No. (%) | 16200 (4.1) | 74 (4.3) | 40 (4.1) |
| Metformin, No. (%) | 10708 (2.7) | 86 (5.0) | 60 (6.2) |
| Glucosamine, No. (%) | 25336 (6.4) | 74 (4.3) | 44 (4.5) |
| Cod liver oil capsule, No. (%) | 21176 (5.3) | 75 (4.3) | 41 (4.2) |

^1^Non-steroidal anti-inflammatory drugs included aspirin, ibuprofen, diclofenac, naproxen, indomethacin, celecoxib, and meloxicam.

^2^Angiotensin converting enzyme inhibitors included captopril, enalapril, lisinopril, fosinopril, ramipril, and quinapril.

^3^Angiotensin II receptor blockers included losartan, candesartan, eprosartan, irbesartan, olmesartan, telmisartan, and valsartan.

^4^Dihydropyridine calcium channel blockers included amlodipine, felodipine, isradipine, nicardipine, and nifedipine.

^5^Beta blockers included acebutolol, atenolol, bisoprolol, carvedilol, labetalol, metoprolol, nadolol, nebivolol, pindolol, and propranolol.

^6^Thiazolidinediones included rosiglitazone, troglitazone, and pioglitazone.

^7^Sulfonylureas included glipizide, glibenclamide, glibornuride, gliclazide, gliquidone, acetohexamide, tolbutamide, chlorpropamide, and tolazamide.

**S3 Table.** Risk Ratios for Baseline Characteristics for Participants with a Minimum of One Positive Covid-19 Test Relative to Participants with No Positive Test

| **Characteristic** | **Relative Risk**  **(95% CI)** | **P Value** | **Adjusted Relative Risk**^4^  **(95% CI)** | **P Value** |
| --- | --- | --- | --- | --- |
| **Baseline and demographic** |  |  |  |  |
| Age* | 1.10 (1.00-1.20) | 0.04 | 1.16 (1.06-1.28) | <0.001 |
| Sex – male vs. female | 1.41 (1.25-1.61) | <0.001 | 1.42 (1.25-1.62) | <0.001 |
| Body-mass index*^,1^ | 1.87 (1.69-2.08) | <0.001 | 1.74 (1.55-1.95) | <0.001 |
| Systolic blood pressure* ­ | 1.03 (1.00-1.07) | 0.04 | 0.99 (0.95-1.02) | 0.45 |
| Race – vs. white |  |  |  |  |
| Asian | 2.28 (1.72-3.01) | <0.001 | 2.13 (1.60-2.85) | <0.001 |
| Black | 3.66 (2.83-4.74) | <0.001 | 2.53 (1.92-3.33) | <0.001 |
| Townsend deprivation index | 1.12 (1.10-1.14) | <0.001 | 1.09 (1.07-1.12) | <0.001 |
| **Social habit** |  |  |  |  |
| Smoking – vs. never |  |  |  |  |
| Previous | 1.40 (1.22-1.60) | <0.001 | 1.33 (1.16-1.53) | <0.001 |
| Current | 1.30 (1.05-1.60) | 0.02 | 1.17 (0.94-1.46) | 0.17 |
| Alcohol use – vs. never |  |  |  |  |
| Once or twice a week | 0.56 (0.45-0.70) | <0.001 | 0.74 (0.59-0.93) | 0.01 |
| Three or four times a week | 0.46 (0.36-0.58) | <0.001 | 0.63 (0.50-0.81) | <0.001 |
| Daily or almost daily | 0.47 (0.37-0.59) | <0.001 | 0.65 (0.50-0.83) | <0.001 |
| **Comorbidity** |  |  |  |  |
| Cancer | 1.15 (0.92-1.44) | 0.21 | 1.23 (0.98-1.54) | 0.08 |
| Diabetes | 2.03 (1.63-2.51) | 0.001 | 1.25 (0.99-1.58) | 0.07 |
| Chronic obstructive pulmonary disease^2^ | 1.96 (1.43-2.68) | <0.001 | 1.64 (1.18-2.27) | 0.003 |
| Asthma | 1.16 (0.97-1.40) | 0.04 | 1.11 (0.92-1.35) | 0.26 |
| Ischemic heart disease^3^ | 2.02 (1.60-2.54) | <0.001 | 1.48 (1.16-1.89) | 0.002 |
| Hypothyroidism | 1.00 (0.74-1.33) | 1.00 | 0.98 (0.71-1.33) | 0.88 |
| Hypercholesterolemia | 1.41 (1.19-1.67) | <0.001 | 1.13 (0.94-1.35) | 0.19 |
| Allergic rhinitis | 0.93 (0.71-1.23) | 0.68 | 1.05 (0.80-1.39) | 0.71 |
| Depression | 1.39 (1.10-1.76) | 0.01 | 1.32 (1.03-1.70) | 0.027 |
| **Serology** |  |  |  |  |
| White blood cell count | 1.03 (1.02-1.04) | <0.001 | 1.03 (1.02-1.04) | <0.001 |
| Red blood cell count | 1.25 (1.05-1.47) | 0.01 | 0.87 (0.73-1.04) | 0.16 |
| Hemoglobin concentration | 1.01 (0.95-1.07) | 0.73 | 0.93 (0.87-1.00) | 0.05 |
| Mean corpuscular volume | 0.98 (0.97-1.00) | 0.03 | 1.00 (0.99-1.02) | 0.75 |
| Mean corpuscular hemoglobin concentration | 0.89 (0.83-0.96) | 0.002 | 0.94 (0.88-1.00) | 0.07 |
| Platelet count | 1.00 (1.00-1.00) | 0.30 | 1.00 (1.00-1.00) | 0.76 |
| Lymphocyte count | 1.03 (1.02-1.04) | <0.001 | 1.03 (1.01-1.05) | 0.008 |
| Monocyte count | 1.17 (1.10-1.24) | <0.001 | 1.16 (1.06-1.27) | 0.002 |
| Neutrophil count | 1.09 (1.05-1.14) | <0.001 | 1.07 (1.02-1.11) | 0.003 |

*Risk ratios are presented per 10 unit increase in risk factor.

^1^Body-mass index is the weight in kilograms divided by the square of the height in meters.

^2^Chronic obstructive pulmonary disease was defined as a diagnosis of emphysema and/or bronchitis.

^3^Ischemic heart disease was categorized as history of myocardial infarction or angina.

^4^Adjusted estimates were controlled for age, sex, body-mass index, systolic blood pressure, race, and Townsend deprivation score.

**S4 Table.** Risk Ratios for Medication Use for Participants with a Minimum of One Positive Covid-19 Test Relative to Participants with No Positive Test

| **Medication Class** | **Relative Risk (95% CI)** | **P Value** | **Adjusted Relative Risk (95% CI)**^8^ | **P Value** |
| --- | --- | --- | --- | --- |
| Non-steroidal anti-inflammatory drug^1^ | 1.19 (1.04-1.37) | 0.02 | 1.06 (0.92-1.23) | 0.41 |
| Angiotensin converting enzyme inhibitor^2^ | 1.92 (1.48-2.48) | <0.001 | 1.48 (1.13-1.93) | 0.004 |
| Angiotensin II receptor blocker^3^ | 1.69 (1.25-2.28) | 0.001 | 1.27 (0.93-1.74) | 0.14 |
| Dihydropyridine calcium channel blocker^4^ | 1.78 (1.43-2.22) | <0.001 | 1.25 (0.99-1.58) | 0.06 |
| Beta blocker^5^ | 1.74 (1.41-2.14) | <0.001 | 1.35 (1.08-1.69) | 0.008 |
| Thiazolidinedione^6^ | 1.50 (0.63-3.61) | 0.39 | 0.69 (0.26-1.87) | 0.47 |
| Sulfonylurea^7^ | 3.00 (2.03-4.42) | <0.001 | 1.74 (1.15-2.61) | 0.008 |
| **Other Common Therapies** |  |  |  |  |
| Acetaminophen | 1.31 (1.12-1.52) | <0.001 | 1.23 (1.05-1.43) | 0.01 |
| Levothyroxine | 1.01 (0.74-1.39) | 0.94 | 0.98 (0.70-1.37) | 0.90 |
| Metformin | 2.38 (1.84-3.09) | <0.001 | 1.30 (0.97-1.73) | 0.08 |
| Glucosamine | 0.70 (0.52-0.95) | 0.02 | 0.75 (0.55-1.03) | 0.07 |
| Cod liver oil capsule | 0.79 (0.57-1.07) | 0.13 | 0.86 (0.63-1.18) | 0.35 |

^1^Non-steroidal anti-inflammatory drugs included aspirin, ibuprofen, diclofenac, naproxen, indomethacin, celecoxib, and meloxicam.

^2^Angiotensin converting enzyme inhibitors included captopril, enalapril, lisinopril, fosinopril, ramipril, and quinapril.

^3^Angiotensin II receptor blockers included losartan, candesartan, eprosartan, irbesartan, olmesartan, telmisartan, and valsartan.

^4^Dihydropyridine calcium channel blockers included amlodipine, felodipine, isradipine, nicardipine, and nifedipine.

^5^Beta blockers included acebutolol, atenolol, bisoprolol, carvedilol, labetalol, metoprolol, nadolol, nebivolol, pindolol, and propranolol.

^6^Thiazolidinediones included rosiglitazone, troglitazone, and pioglitazone.

^7^Sulfonylureas included glipizide, glibenclamide, glibornuride, gliclazide, gliquidone, acetohexamide, tolbutamide, chlorpropamide, and tolazamide.

^8^Adjusted estimates were controlled for age, sex, body-mass index, systolic blood pressure, race, and Townsend deprivation score.

**S5 Table.** Characteristics of Covid-19 Positive Inpatients and Outpatients

| **Characteristic** | **Covid-19 Positive Outpatients**  **(N=229)** | **Covid-19 Positive Inpatients**  **(N=739)** |
| --- | --- | --- |
| **Baseline and demographic** |  |  |
| Age, mean (SD), y | 55.1 (9.8) | 57.6 (8.8) |
| Male, No. (%) | 105 (45.9) | 416 (56.3) |
| Body-mass index, mean (SD)* | 28.7 (5.5) | 29.2 (5.4) |
| Systolic blood pressure, mean (SD), mmHg | 139.3 (20.7) | 141.4 (20.7) |
| Race, No. (%) |  |  |
| White | 191 (83.4) | 648 (87.9) |
| Asian | 12 (5.2) | 40 (5.4) |
| Black | 23 (10.0) | 38 (5.2) |
| Townsend deprivation index, mean (SD) | -0.2 (3.6) | -0.2 (3.4) |
| **Social habit** |  |  |
| Smoking, No. (%) |  |  |
| Never | 126 (55.0) | 329 (44.6) |
| Previous | 82 (35.8) | 316 (42.9) |
| Current | 21 (9.2) | 84 (11.4) |
| Alcohol use, No. (%) |  |  |
| Never | 33 (14.4) | 93 (12.6) |
| Once or twice a week | 60 (26.2) | 177 (24.0) |
| Three or four times a week | 35 (15.3) | 140 (19.0) |
| Daily or almost daily | 34 (14.8) | 124 (16.8) |
| **Comorbidity** |  |  |
| Cancer, No. (%) | 22 (9.6) | 64 (8.7) |
| Diabetes, No. (%) | 16 (7.0) | 75 (10.2) |
| Chronic obstructive pulmonary disease, No. (%)^1^ | 8 (3.5) | 32 (4.3) |
| Asthma, No. (%) | 32 (14.0) | 96 (13.0) |
| Ischemic heart disease, No. (%)^2^ | 15 (6.6) | 62 (8.4) |
| Hypothyroidism, No. (%) | 14 (6.1) | 33 (4.5) |
| Hypercholesterolemia, No. (%) | 26 (11.4) | 132 (17.9) |
| Allergic rhinitis, No. (%) | 14 (6.1) | 40 (5.4) |
| Depression, No. (%) | 16 (7.0) | 58 (7.8) |
| **Serology** |  |  |
| White blood cell count, mean (SD) | 7.0 (2.0) | 7.4 (4.9) |
| Red blood cell count, mean (SD) | 4.5 (0.5) | 4.6 (0.4) |
| Hemoglobin concentration, mean (SD) | 14.1 (1.5) | 14.2 (1.4) |
| Mean corpuscular volume, mean (SD) | 90.4 (5.5) | 90.7 (5.2) |
| Mean corpuscular hemoglobin concentration, mean (SD) | 34.3 (1.0) | 34.4 (1.0) |
| Platelet count, mean (SD) | 251.9 (66.5) | 249.5 (62.1) |
| Lymphocyte count, mean (SD) | 2.0 (1.4) | 2.2 (4.5) |
| Monocyte count, mean (SD) | 0.5 (0.2) | 0.5 (0.4) |
| Neutrophil count, mean (SD) | 4.3 (1.4) | 4.4 (1.5) |

*Body-mass index is the weight in kilograms divided by the square of the height in meters.

^1^Chronic obstructive pulmonary disease was defined as a diagnosis of emphysema and/or bronchitis.

^2^Ischemic heart disease was categorized as history of myocardial infarction or angina.

**S6 Table.** Frequency of Medication Use in Covid-19 Positive Inpatients and Outpatients

| **Medication Class** | **Covid-19 Positive Outpatients**  **(N=229)** | **Covid-19 Positive Inpatients**  **(N=739)** |
| --- | --- | --- |
| Non-steroidal anti-inflammatory drug, No. (%)^1^ | 65 (28.4) | 219 (29.6) |
| Angiotensin converting enzyme inhibitor, No. (%)^2^ | 14 (6.1) | 48 (6.5) |
| Angiotensin II receptor blocker, No. (%)^3^ | 11 (4.8) | 34 (4.6) |
| Dihydropyridine calcium channel blocker, No. (%)^4^ | 17 (7.4) | 70 (9.5) |
| Beta blocker, No. (%)^5^ | 13 (5.7) | 83 (11.2) |
| Thiazolidinedione, No. (%)^6^ | 1 (0.4) | 4 (0.5) |
| Sulfonylurea, No. (%)^7^ | 1 (0.4) | 25 (3.4) |
| **Other Common Therapies** |  |  |
| Acetaminophen, No. (%) | 50 (21.8) | 169 (22.9) |
| Levothyroxine, No. (%) | 12 (5.2) | 28 (3.8) |
| Metformin, No. (%) | 10 (4.4) | 50 (6.8) |
| Glucosamine, No. (%) | 5 (2.2) | 39 (5.3) |
| Cod liver oil capsule, No. (%) | 9 (3.9) | 32 (4.3) |

^1^Non-steroidal anti-inflammatory drugs included aspirin, ibuprofen, diclofenac, naproxen, indomethacin, celecoxib, and meloxicam.

^2^Angiotensin converting enzyme inhibitors included captopril, enalapril, lisinopril, fosinopril, ramipril, and quinapril.

^3^Angiotensin II receptor blockers included losartan, candesartan, eprosartan, irbesartan, olmesartan, telmisartan, and valsartan.

^4^Dihydropyridine calcium channel blockers included amlodipine, felodipine, isradipine, nicardipine, and nifedipine.

^5^Beta blockers included acebutolol, atenolol, bisoprolol, carvedilol, labetalol, metoprolol, nadolol, nebivolol, pindolol, and propranolol.

^6^Thiazolidinediones included rosiglitazone, troglitazone, and pioglitazone.

^7^Sulfonylureas included glipizide, glibenclamide, glibornuride, gliclazide, gliquidone, acetohexamide, tolbutamide, chlorpropamide, and tolazamide.

**S7 Table.** Odds Ratios for Baseline Characteristics for Inpatient Covid-19 Relative to Outpatient Disease

| **Characteristic** | **Odds Ratio (95% CI)** | **P Value** |
| --- | --- | --- |
| **Baseline and demographic** |  |  |
| Age* | 1.39 (1.19-1.63) | <0.001 |
| Sex – male vs. female | 1.52 (1.13-2.05) | 0.006 |
| Body-mass index*^,1^ | 1.17 (0.89-1.52) | 0.26 |
| Systolic blood pressure* ­ | 1.06 (0.99-1.14) | 0.10 |
| Race – vs. white |  |  |
| Asian | 0.98 (0.51-1.91) | 1.00 |
| Black | 0.49 (0.28-0.84) | 0.01 |
| Mean Townsend deprivation index | 1.02 (0.98-1.07) | 0.29 |
| **Social habit** |  |  |
| Smoking – vs. never |  |  |
| Previous | 1.48 (1.07-2.03) | 0.02 |
| Current | 1.53 (0.91-2.58) | 0.11 |
| Alcohol use – vs. never |  |  |
| Once or twice a week | 1.05 (0.64-1.71) | 0.90 |
| Three or four times a week | 1.42 (0.82-2.44) | 0.21 |
| Daily or almost daily | 1.29 (0.75-2.24) | 0.40 |
| **Comorbidity** |  |  |
| Cancer | 0.89 (0.54-1.48) | 0.69 |
| Diabetes | 1.51 (0.86-2.65) | 0.16 |
| Chronic obstructive pulmonary disease^2^ | 1.25 (0.57-2.75) | 0.71 |
| Asthma | 0.91 (0.60-1.41) | 0.74 |
| Ischemic heart disease^3^ | 1.31 (0.73-2.34) | 0.41 |
| Hypothyroidism | 0.72 (0.38-1.37) | 0.30 |
| Hypercholesterolemia | 1.70 (1.08-2.66) | 0.02 |
| Allergic rhinitis | 0.88 (0.47-1.65) | 0.74 |
| Depression | 1.13 (0.64-2.01) | 0.78 |

*Risk ratios are presented per 10 unit increase in risk factor.

^1^Body-mass index is the weight in kilograms divided by the square of the height in meters.

^2^Chronic obstructive pulmonary disease was defined as a diagnosis of emphysema and/or bronchitis.

^3^Ischemic heart disease was categorized as history of myocardial infarction or angina.

**S8 Table.** Odds Ratios for Medication Use for Inpatient Covid-19 Relative to Outpatient Disease

| **Medication Class** | **Odds Ratio (95% CI)** | **P Value** |
| --- | --- | --- |
| Non-steroidal anti-inflammatory drug^1^ | 1.06 (0.77-1.47) | 0.74 |
| Angiotensin converting enzyme inhibitor^2^ | 1.07 (0.58-1.97) | 1.00 |
| Angiotensin II receptor blocker^3^ | 0.96 (0.48-1.92) | 0.86 |
| Dihydropyridine calcium channel blocker^4^ | 1.30 (0.75-2.27) | 0.43 |
| Beta blocker^5^ | 2.10 (1.15-3.85) | 0.02 |
| Thiazolidinedione^6^ | 1.24 (0.14-11.16) | 1.00 |
| Sulfonylurea^7^ | 7.98 (1.08-59.24) | 0.02 |
| **Other Common Therapies** |  |  |
| Acetaminophen | 1.06 (0.74-1.52) | 0.79 |
| Levothyroxine | 0.71 (0.36-1.42) | 0.34 |
| Metformin | 1.58 (0.79-3.19) | 0.21 |
| Glucosamine | 2.50 (0.97-6.41) | 0.07 |
| Cod liver oil capsule | 1.11 (0.52-2.35) | 1.00 |

^1^Non-steroidal anti-inflammatory drugs included aspirin, ibuprofen, diclofenac, naproxen, indomethacin, celecoxib, and meloxicam.

^2^Angiotensin converting enzyme inhibitors included captopril, enalapril, lisinopril, fosinopril, ramipril, and quinapril.

^3^Angiotensin II receptor blockers included losartan, candesartan, eprosartan, irbesartan, olmesartan, telmisartan, and valsartan.

^4^Dihydropyridine calcium channel blockers included amlodipine, felodipine, isradipine, nicardipine, and nifedipine.

^5^Beta blockers included acebutolol, atenolol, bisoprolol, carvedilol, labetalol, metoprolol, nadolol, nebivolol, pindolol, and propranolol.

^6^Thiazolidinediones included rosiglitazone, troglitazone, and pioglitazone.

^7^Sulfonylureas included glipizide, glibenclamide, glibornuride, gliclazide, gliquidone, acetohexamide, tolbutamide, chlorpropamide, and tolazamide.

**S9 Table.** Risk Ratios for Census Measures for Participants with a Minimum of One Positive Covid-19 Test Relative to Participants with No Positive Test

| **Category^1^** | **Adjusted Relative Risk^2^ (95% CI)** | **P Value** |
| --- | --- | --- |
| **Usual Resident Population** |  |  |
| Total Individuals | 1.00 (0.94-1.06) | 0.98 |
| Individuals per Hectare | 1.14 (1.08-1.20) | <0.001 |
| **Age Structure** |  |  |
| Individuals 5 to 7 years | 1.04 (0.98-1.10) | 0.21 |
| Individuals 30 to 44 years | 1.01 (0.94-1.07) | 0.8 |
| Individuals 45 to 59 years | 0.89 (0.83-0.95) | <0.001 |
| Individuals 75 to 84 years | 0.93 (0.87-0.99) | 0.03 |
| Median Age | 0.89 (0.84-0.95) | <0.001 |
| **Economic Activity** |  |  |
| Individuals self employed | 0.84 (0.78-0.90) | <0.001 |
| Individuals unemployed | 1.19 (1.12-1.25) | <0.001 |
| Individuals retired | 0.91 (0.85-0.975) | 0.007 |
| **Ethnic Group^3^** |  |  |
| White Individuals | 0.90 (0.85-0.96) | 0.001 |
| Multiple Ethnicities Individuals | 1.14 (1.08-1.20) | <0.001 |
| Black Individuals | 1.12 (1.07-1.17) | <0.001 |
| **Health and Provisions of Unpaid Care** |  |  |
| Individuals Aged 16 to 64 Activities Limited A Lot | 1.18 (1.11-1.24) | <0.001 |
| Individuals Very Good Health Measures | 0.94 (0.87-1.00) | 0.05 |
| Individuals Good Health Measures | 0.98 (0.92-1.05) | 0.60 |
| Individuals Bad Health Measures | 1.17 (1.10-1.24) | <0.001 |
| Individuals Very Bad Health Measures | 1.18 (1.12-1.24) | <0.001 |
| Individuals Provide 1 to 19 Hours Unpaid Care | 0.85 (0.80-0.91) | 0.79 |
| Individuals Provide 20 to 49 Hours Unpaid Care | 1.11 (1.05-1.18) | <0.001 |
| Individuals Provide 50 or More Hours Unpaid Care | 1.11 (1.05-1.18) | <0.001 |
| **Household Composition** |  |  |
| Individuals Lone Parents | 1.18 (1.12-1.25) | <0.001 |
| Individuals in Other Household Type with Dependent Children | 1.08 (1.02-1.13) | 0.01 |
| **Living Arrangements** |  |  |
| Individuals Living Not in Couple | 1.16 (1.09-1.23) | <0.001 |
| Individuals Separated but Married | 1.15 (1.08-1.22) | <0.001 |
| **Qualifications** |  |  |
| Individuals with No Qualification | 1.14 (1.07-1.32) | <0.001 |
| Individuals with Level 2 Qualification | 0.95 (0.90-1.02) | 0.18 |
| Individuals with Level 4 Qualification | 0.89 (0.83-0.95) | <0.001 |
| **Tenure** |  |  |
| Individuals Own Home | 0.83 (0.78-0.88) | 0.002 |
| Individuals Socially Rent | 1.18 (1.12-1.24) | <0.001 |
| Individuals Rent from Other | 1.13 (1.07-1.19) | <0.001 |

^1^Full definitions of each census category is described on the UK census website (<https://www.nomisweb.co.uk/census/2011/key_statistics_uk>).

^2^Risk ratios were adjusted for age, sex, body-mass index, systolic blood pressure, and race. Each census feature described was first standardized to have a mean value of zero and standard deviation of one.

^3^Any significant ethnic group association does not in any way indicate the people of the described ethnicity mechanistically cause the risk, but rather simply live in a locale where socioeconomic and healthcare factors associate with increased risk.

**S10 Table.** Odds Ratios for Census Measures for Inpatient Covid-19 Relative to Outpatient Disease

| **Category^1^** | **Odds Ratio^2^ (95% CI)** | **P Value** |
| --- | --- | --- |
| **Usual Resident Population** |  |  |
| Total Individuals | 1.13 (0.96-1.33) | 0.15 |
| Individuals per Hectare | 0.918 (0.79-1.07) | 0.27 |
| **Age Structure** |  |  |
| Individuals 5 to 7 years | 0.89 (0.77-1.03) | 0.13 |
| Individuals 30 to 44 years | 0.96 (0.82-1.13) | 0.65 |
| Individuals 45 to 59 years | 1.26 (1.08-1.47) | 0.004 |
| Individuals 75 to 84 years | 1.17 (0.99-1.38) | 0.06 |
| Median Age | 1.21 (1.03-1.42) | 0.02 |
| **Economic Activity** |  |  |
| Individuals self employed | 0.95 (0.82-1.11) | 0.54 |
| Individuals unemployed | 1.02 (0.87-1.20) | 0.77 |
| Individuals retired | 1.33 (1.12-1.58) | 0.001 |
| **Ethnic Group^3^** |  |  |
| White Individuals | 1.18 (1.00-1.40) | 0.04 |
| Multiple Ethnicities Individuals | 0.80 (0.68-0.93) | 0.003 |
| Caribbean Individuals | 0.83 (0.71-0.97) | 0.01 |
| **Health and Provisions of Unpaid Care** |  |  |
| Individuals Aged 16 to 64 Activities Limited A Lot | 1.17 (0.99-1.38) | 0.06 |
| Individuals Very Good Health Measures | 1.04 (0.89-1.22) | 0.63 |
| Individuals Good Health Measures | 1.09 (0.93-1.27) | 0.30 |
| Individuals Bad Health Measures | 1.18 (1.00-1.38) | 0.05 |
| Individuals Very Bad Health Measures | 1.11 (0.95-1.30) | 0.20 |
| Individuals Provide 1 to 19 Hours Unpaid Care | 1.22 (1.04-1.44) | 0.01 |
| Individuals Provide 20 to 49 Hours Unpaid Care | 1.24 (1.06-1.46) | 0.006 |
| Individuals Provide 50 or More Hours Unpaid Care | 1.29 (1.10-1.51) | 0.002 |
| **Household Composition** |  |  |
| Individuals Lone Parents | 0.96 (0.82-1.12) | 0.59 |
| Individuals in Other Household Type with Dependent Children | 1.02 (0.86-1.21) | 0.83 |
| **Living Arrangements** |  |  |
| Individuals Living Not in Couple | 1.05 (0.90-1.24) | 0.57 |
| Individuals Separated but Married | 0.96 (0.82-1.13) | 0.63 |
| **Qualifications** |  |  |
| Individuals with No Qualification | 1.17 (0.99-1.37) | 0.05 |
| Individuals with Level 2 Qualification | 1.18 (1.01-1.39) | 0.03 |
| Individuals with Level 4 Qualification | 0.96 (0.82-1.13) | 0.64 |
| **Tenure** |  |  |
| Individuals Own Home | 1.22 (1.04-1.43) | 0.01 |
| Individuals Socially Rent | 0.98 (0.84-1.14) | 0.80 |
| Individuals Rent from Other | 0.99 (0.86-1.16) | 0.99 |

^1^Full definitions of each census category is described on the UK census website (<https://www.nomisweb.co.uk/census/2011/key_statistics_uk>).

^2^Odds ratios were adjusted for age, sex, body-mass index, systolic blood pressure, and race. Each census feature described was first standardized to have a mean value of zero and standard deviation of one.

^3^Any significant ethnic group association does not in any way indicate the people of the described ethnicity mechanistically cause the risk, but rather simply live in a locale where socioeconomic and healthcare factors associate with increased risk.

**S11 Table.** Odds Ratios for Blood Type for Participants with a Minimum of One Positive Covid-19 Test Relative to Participants with No Positive Test

| Blood Type (%)* | Blood Type Compared to | Odds Ratio (95% CI) | P Value |
| --- | --- | --- | --- |
| A (43.8) | B | 1.08 (0.87-1.35) | 0.50 |
|  | O | 1.16 (1.01-1.33) | 0.03 |
| B (9.5) | A | 0.93 (0.74-1.16) | 0.50 |
|  | O | 1.08 (0.86-1.34) | 0.51 |
| O (43.1) | A | 0.86 (0.75-0.99) | 0.03 |
|  | B | 0.93 (0.74-1.16) | 0.51 |

*The remaining population with genotypes available were either an ambiguous blood type or had missing alleles that prevented a blood type inference.

**S1 Fig.** Location of UK Biobank Testing Centers


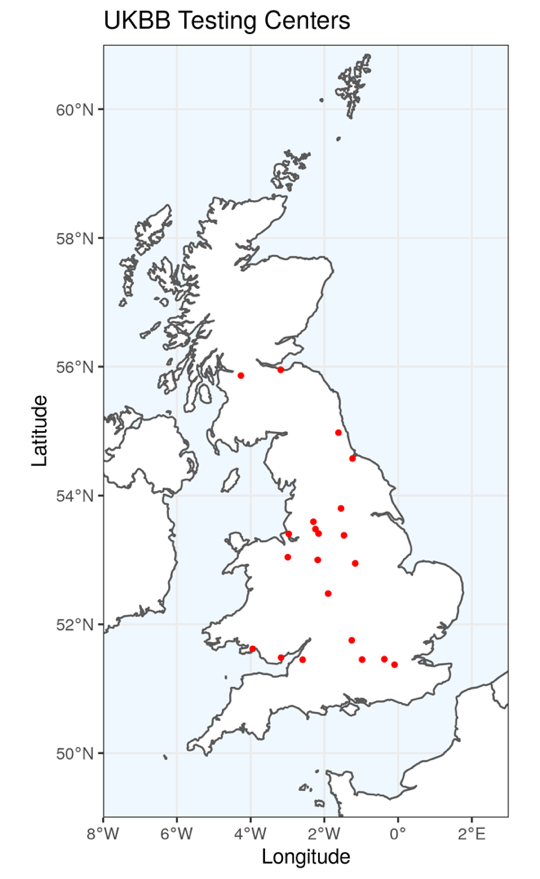


This map was derived from *Natural Earth,* which falls under the Creative Commons Public Domain license.

**S2 Fig.** Location and Status of Individuals Tested for Covid-19


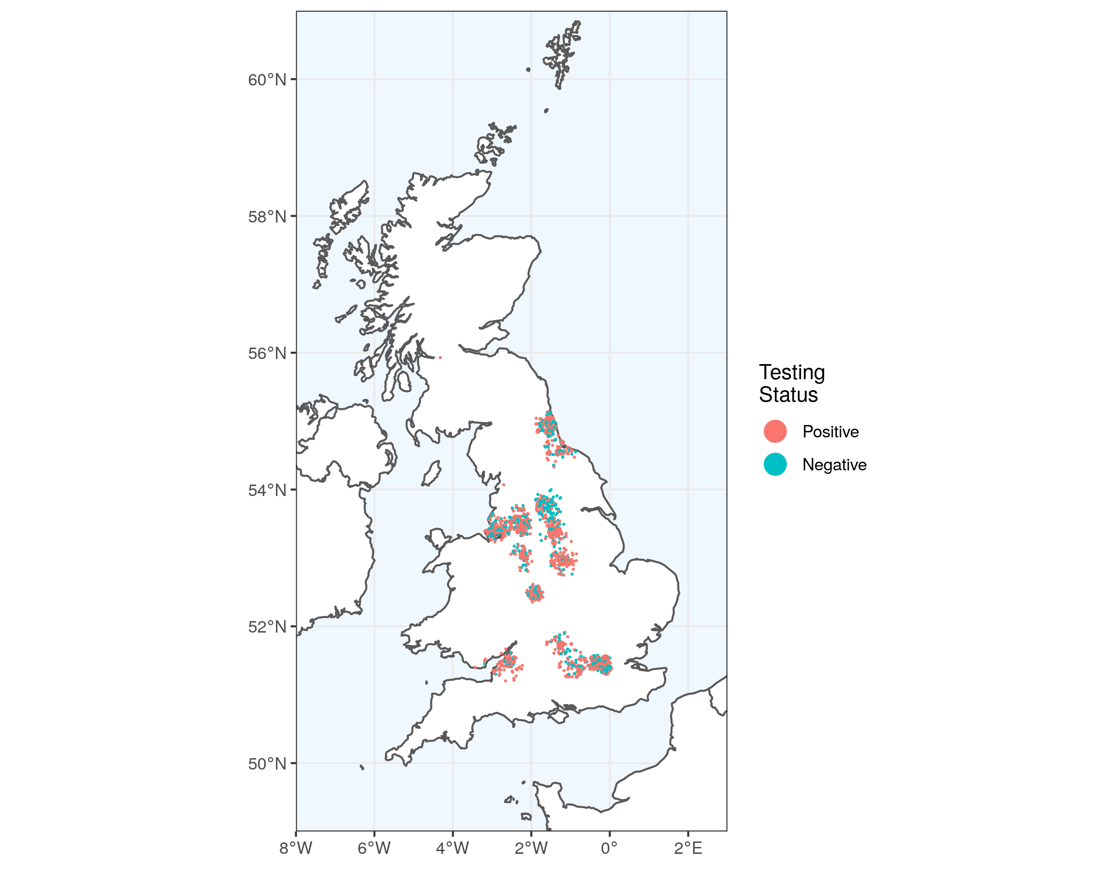


This map was derived from *Natural Earth,* which falls under the Creative Commons Public Domain license.

**S3 Fig.** First Two Principal Components Used in Genetic QC


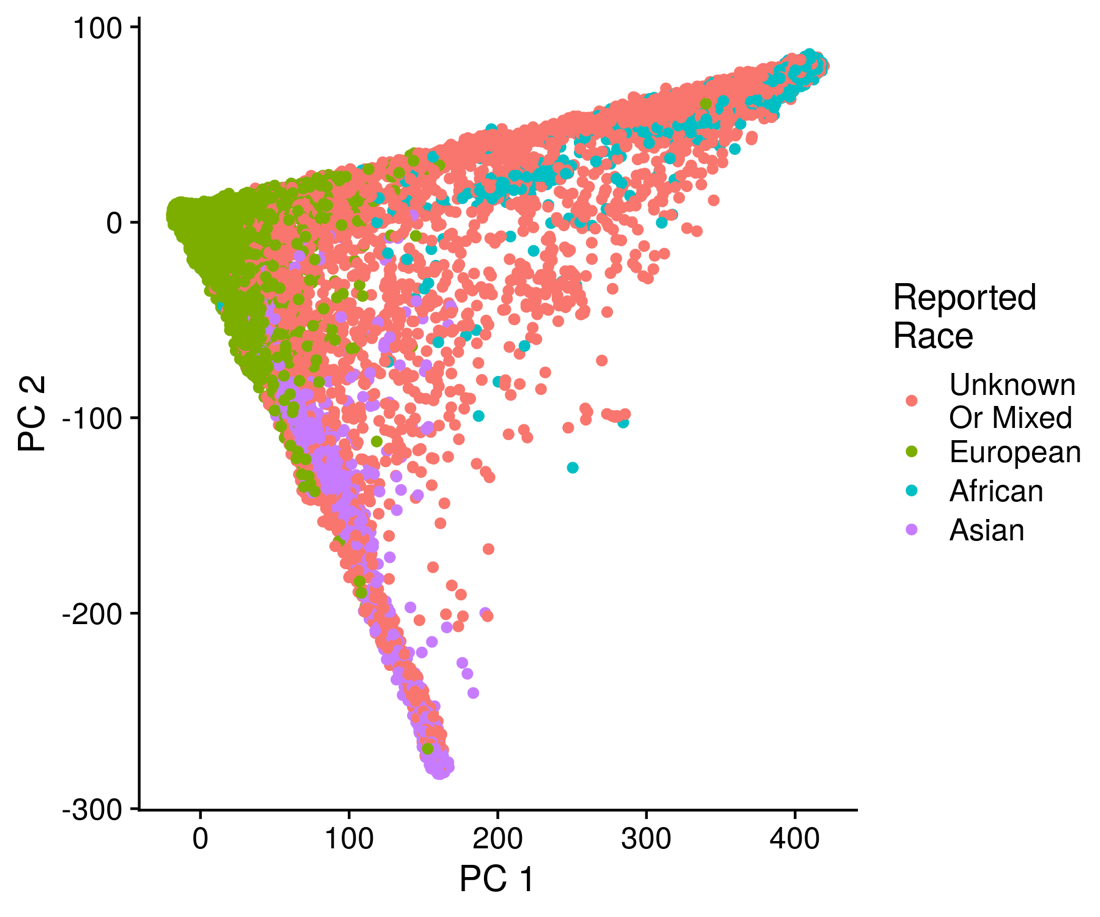


Principal components analysis of UK Biobank genetic data was completed by personnel working at the UK Biobank, with procedures described in the document “Genotyping and quality control of UK Biobank, a large-scale, extensively phenotyped prospective resource”. The first two principal components from this analysis are shown, colored by each individual’s self-described race. From the full 40 principal components ancestry groups were determined, as described in the Methods Section.

**S4 Fig.** European Ancestry Genome-wide Association Analysis of Covid-19 Positive Status


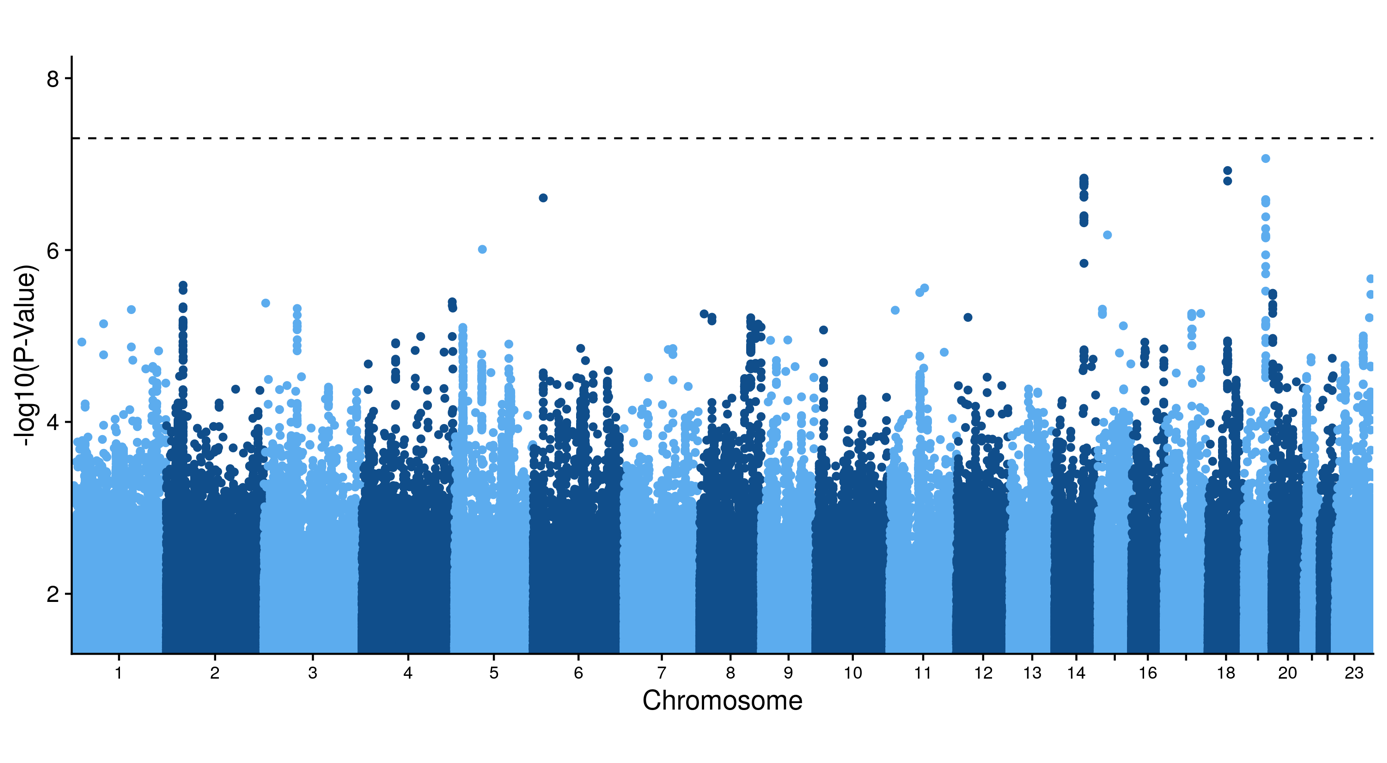


The genome-wide association analysis was conducted on UK Biobank individuals of European ancestry residing within England, comparing individuals that tested positive once to the remaining population. There were 375,921 controls (did not test positive) and 846 cases (tested positive). In total, 9,250,219 imputed variants were tested through logistic-Firth hybrid regression corrected for age, sex, and the first 10 genetic principal components. Variants were removed if their missing rate was above 10%, Hardy-Weinberg Equilibrium Test P-Value < 1 x 10^-50^, or the allele frequency was < 0.001. The chromosomes are ordered numerically from 1 on the left to X (chromosome 23) on the right, with each progressive color change representing a different chromosome. The significance level employed was P = 5 x 10^-8^. Only variants with P < 0.05 are shown in this plot.

**S5 Fig.** African Ancestry Genome-wide Association Analysis of Covid-19 Positive Status


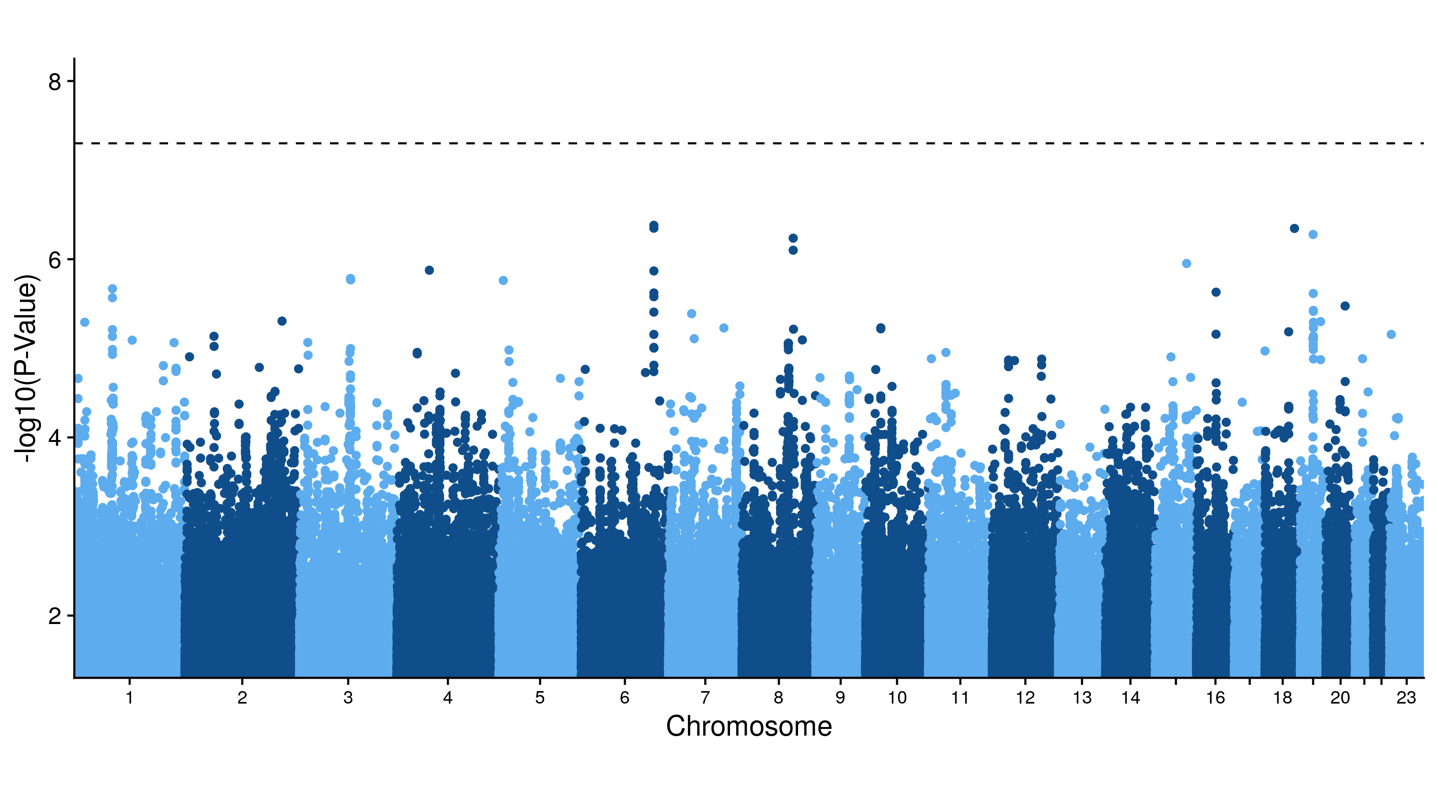


The genome-wide association analysis was conducted on UK Biobank individuals of African ancestry, comparing individuals that tested positive once to the remaining population. There were 8,214 controls (did not test positive) and 66 cases (tested positive). . In total, 5,074,382 imputed variants were tested through logistic-Firth hybrid regression corrected for age, sex, and the first 10 genetic principal components. Variants were removed if their missing rate was above 10%, Hardy-Weinberg Equilibrium Test P-Value was < 1 x 10^-50^, or the allele frequency was < 0.05. The chromosomes are ordered numerically from 1 on the left to X (chromosome 23) on the right, with each progressive color change representing a different chromosome. The significance level employed was P = 5 x 10^-8^. Only variants with P < 0.05 are shown in this plot.

**S6 Fig.** Asian Ancestry Genome-wide Association Analysis of Covid-19 Positive Status


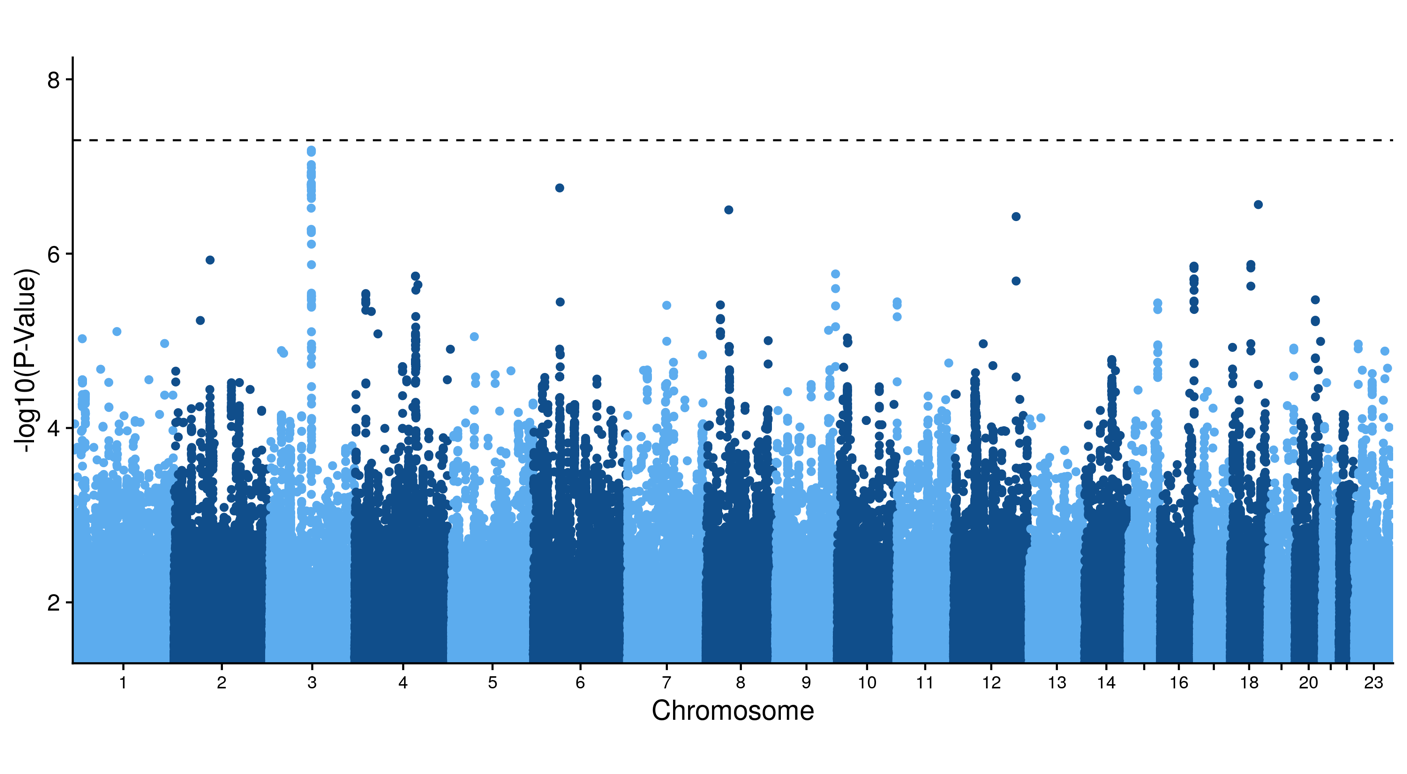


The genome-wide association analysis was conducted on UK Biobank individuals of Asian ancestry, comparing individuals that tested positive once to the remaining population. There were 11,960 controls (did not test positive) and 56 cases (tested positive). In total, 6,029,896 imputed variants were tested through logistic-Firth hybrid regression corrected for age, sex, and the first 10 genetic principal components. Variants were removed if their missing rate was above 10%, Hardy-Weinberg Equilibrium Test P-Value was < 1 x 10^-50^, or the allele frequency was < 0.05. The chromosomes are ordered numerically from 1 on the left to X (chromosome 23) on the right, with each progressive color change representing a different chromosome. The significance level employed was P = 5 x 10^-8^. Only variants with P < 0.05 are shown in this plot.

**S7 Fig.** Cross-Ancestry HLA specific Analysis of Covid-19 Positive Status


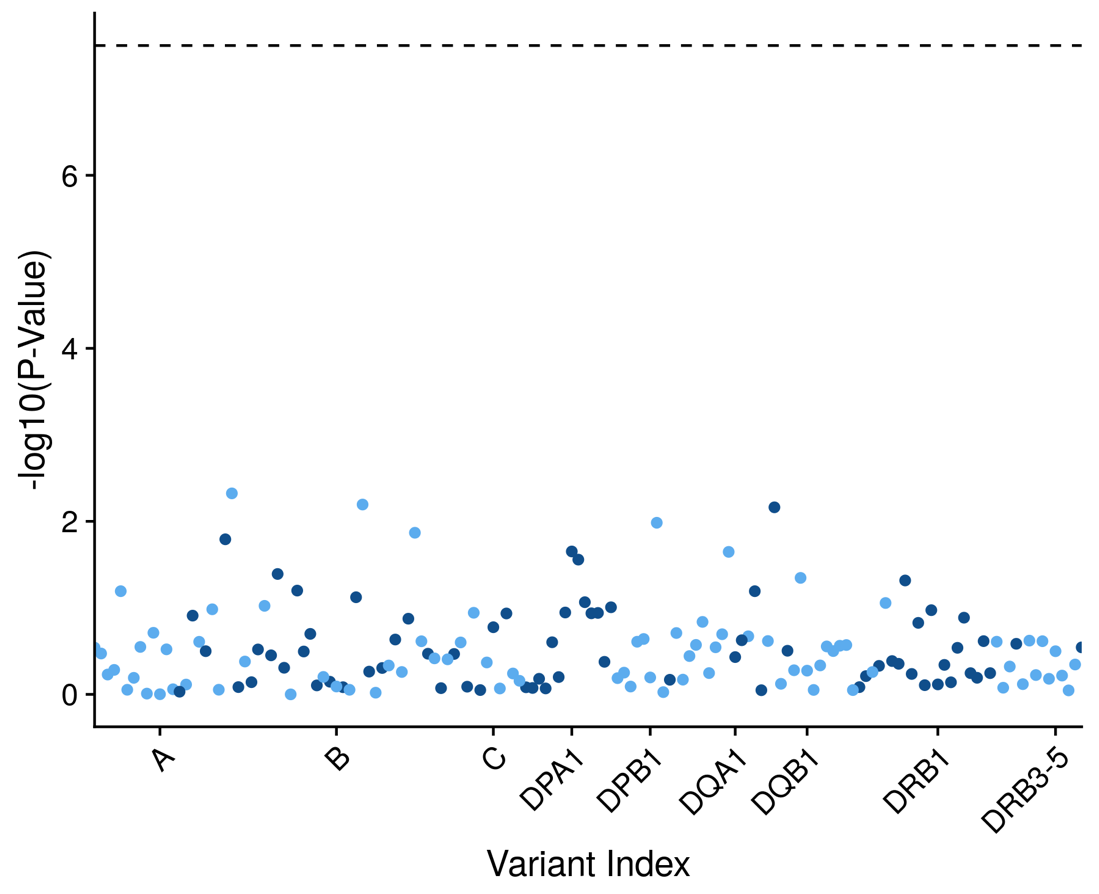


The HLA specific analysis was conducted on HLA allele probabilities determined through HLA*IMP:02. Only HLA alleles where more than one percent of the individuals under analysis contained a nonzero value were tested. Each HLA allele was tested through logistic-Firth hybrid regression adjusted for age, sex and the first 10 genetic principal components. Regression results that converged were meta-analyzed using the weighted Z option in PLINK. If an allele was only tested in a single ancestry group it was left in the meta-analysis results unchanged.

**S8 Fig.** Cross-Ancestry Genome-wide Association Nested Analysis of Inpatient Status for Participants with Any Positive Covid-19 Test


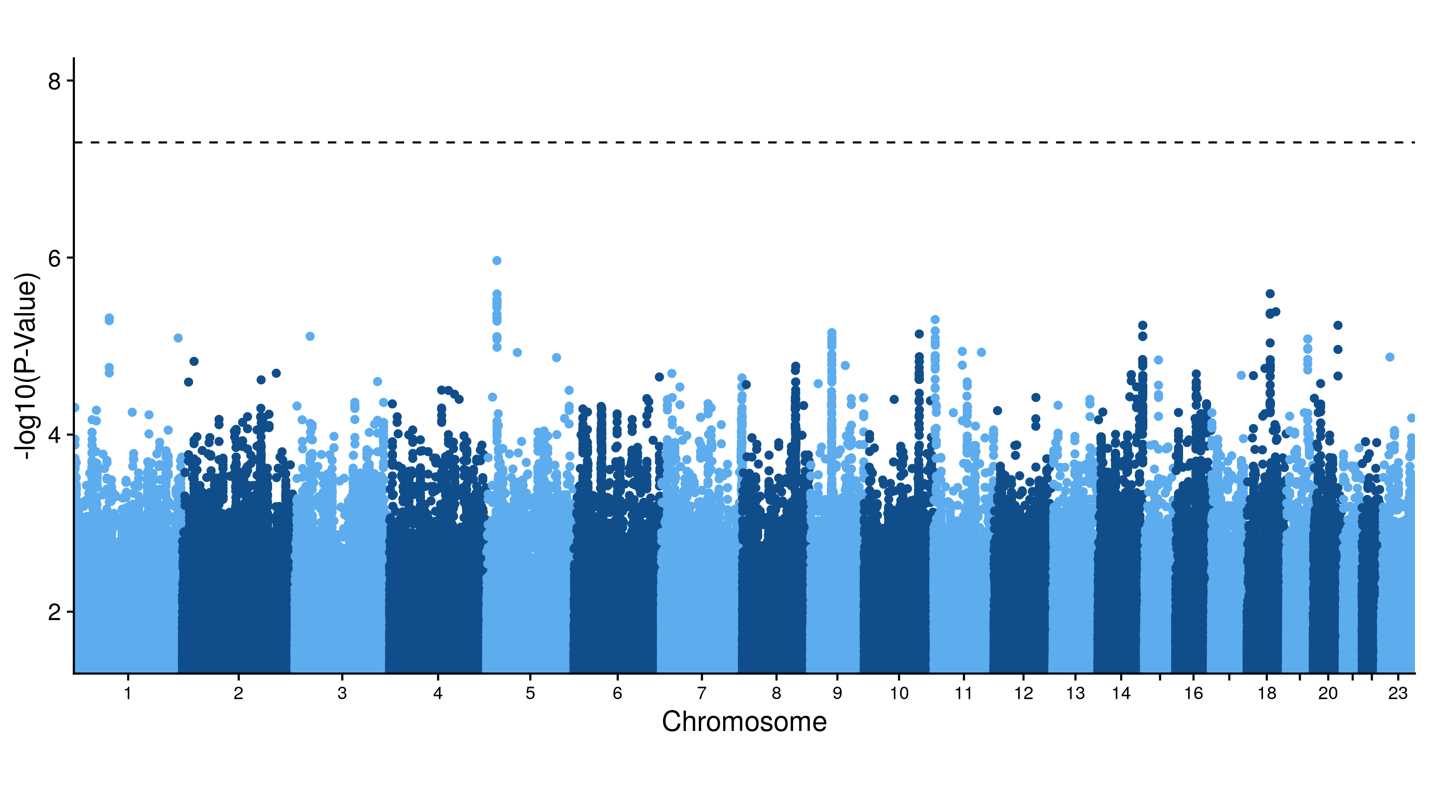


The genome-wide association analysis was conducted on UK Biobank individuals of all ancestry groups, from the population that tested positive once comparing inpatients to outpatients. There were 193, 24 and 12 controls (outpatients) and 653, 42 and 44 cases (inpatients) of European, African and Asian ancestry groups, respectively. In total, 9,303,138 imputed variants were tested through logistic-Firth hybrid regression corrected for age, sex, and the first 10 genetic principal components. Variants were removed if their missing rate was above 10%, Hardy-Weinberg Equilibrium Test P-Value was < 1 x 10^-50^, or the allele frequency was < 0.001. The final p-values were the product of a meta-analysis using weighted Z option in PLINK. The chromosomes are ordered numerically from 1 on the left to X (chromosome 23) on the right, with each progressive color change representing a different chromosome. The significance level employed was P = 5 x 10^-8^. Only variants with P < 0.05 are shown in this plot.

**S9 Fig.** Cross-Ancestry HLA specific Nested Analysis of Inpatient Status for Participants with Any Positive Covid-19 Test


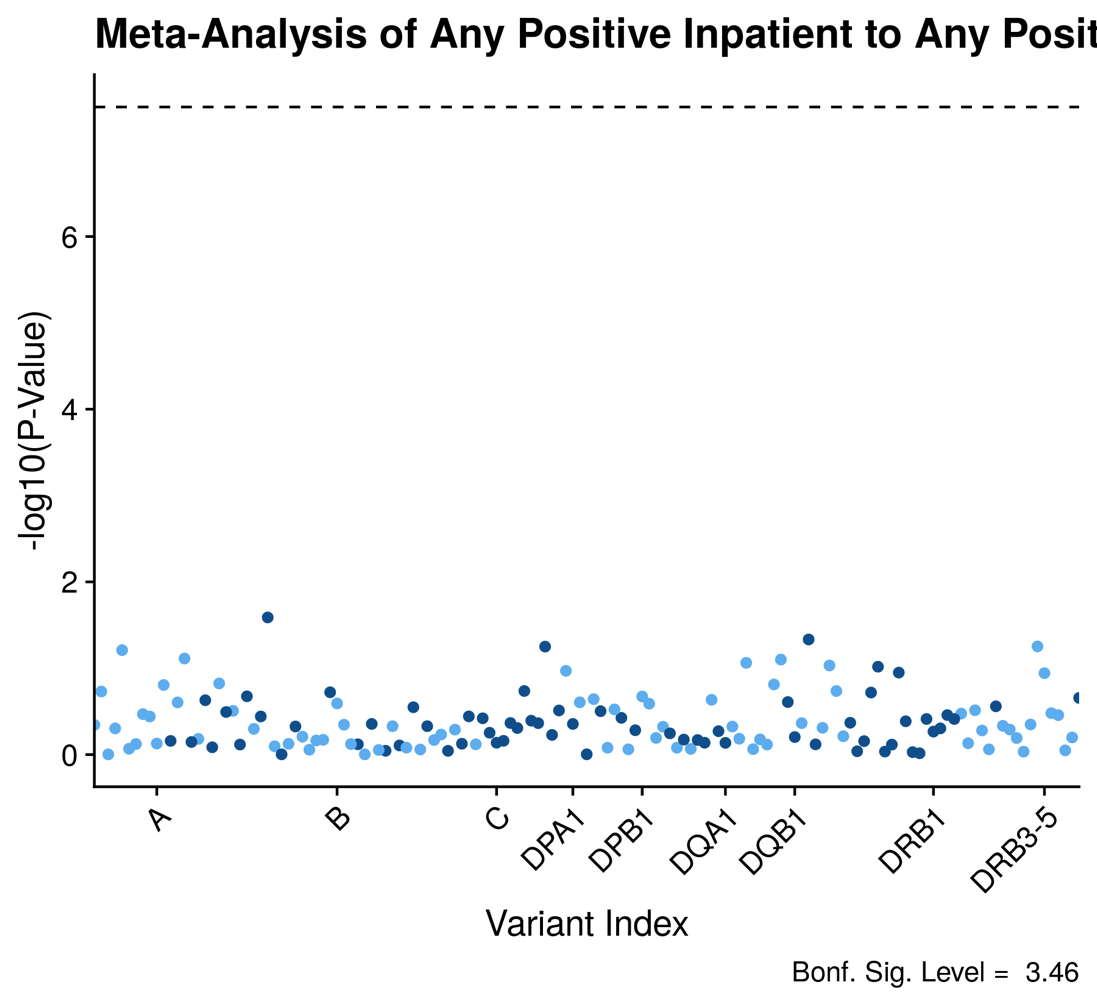


The HLA specific analysis was conducted on HLA allele probabilities determined through HLA*IMP:02. Only HLA alleles where more than one percent of the individuals under analysis contained a nonzero value were tested. Each HLA allele was tested through logistic-Firth hybrid regression adjusted for age, sex and the first 10 genetic principal components. Regression results that converged were meta-analyzed using the weighted Z option in PLINK. If an allele was only tested in a single ancestry group, it was left in the meta-analysis results unchanged.
